# Quantitative analysis of transcription factor binding and expression using calling cards reporter arrays

**DOI:** 10.1093/nar/gkaa141

**Published:** 2020-03-05

**Authors:** Jiayue Liu, Christian A Shively, Robi D Mitra

**Affiliations:** 1 Department of Genetics, Washington University School of Medicine in St. Louis, St. Louis, MO 63108, USA; 2 The Edison Family Center for Genome Sciences & Systems Biology, Washington University School of Medicine in St. Louis, St. Louis, MO 63108, USA; 3 McDonnell Genome Institute, Washington University School of Medicine in St. Louis, St. Louis, MO 63108, USA

## Abstract

We report a tool, Calling Cards Reporter Arrays (CCRA), that measures transcription factor (TF) binding and the consequences on gene expression for hundreds of synthetic promoters in yeast. Using Cbf1p and MAX, we demonstrate that the CCRA method is able to detect small changes in binding free energy with a sensitivity comparable to *in vitro* methods, enabling the measurement of energy landscapes *in vivo*. We then demonstrate the quantitative analysis of cooperative interactions by measuring Cbf1p binding at synthetic promoters with multiple sites. We find that the cooperativity between Cbf1p dimers varies sinusoidally with a period of 10.65 bp and energetic cost of 1.37 K_B_T for sites that are positioned ‘out of phase’. Finally, we characterize the binding and expression of a group of TFs, Tye7p, Gcr1p and Gcr2p, that act together as a ‘TF collective’, an important but poorly characterized model of TF cooperativity. We demonstrate that Tye7p often binds promoters without its recognition site because it is recruited by other collective members, whereas these other members require their recognition sites, suggesting a hierarchy where these factors recruit Tye7p but not vice versa. Our experiments establish CCRA as a useful tool for quantitative investigations into TF binding and function.

## INTRODUCTION

Transcription factors (TFs) recognize and bind to specific sequences in regulatory DNA, called TF binding sites (TFBSs), and these events ultimately define the transcriptional programs that cells execute as they proliferate, develop, and respond to their environments (1–3). The principles that govern how TFs select functional binding sites *in vivo* are not well understood. For example, the *in vivo* occupancies of TFs cannot be predicted solely from their DNA binding preferences measured *in vitro*. Many TFs bind to only a small fraction of high-scoring TFBS in the genome, and, conversely, TF binding is often observed at loci without a nearby TFBS (4–6). Explaining the binding of paralogous TFs is a related outstanding problem, as such factors often have nearly identical *in vitro* DNA binding preferences but regulate diverse sets of target genes and perform different cellular functions, even when expressed at the same time and in the same cell (7–9). Finally, the relationship between TF binding and the resulting transcriptional consequences is also unclear, as it is difficult to predict whether a TF binding event will have any effect on the expression of a nearby gene or the directionality of such a change. Part of the reason for these difficulties is that TFs appear to act in a highly complex manner. Many TFs bind cooperatively (10–15), and we are far from having a complete description of which TFs interact with one another, or how they select their binding sites when they do interact. Even TFs that bind DNA independently may recruit transcriptional machinery in a combinatorial fashion after they bind to influence gene expression (16). Therefore, we need new experimental tools to study gene regulation that are quantitative, allow for the rapid analysis of many user-specified regulatory sequences, and can be easily multiplexed to study a number of different TFs.

High throughput methods such as Sort-Seq (17,18) and Massively Parallel Reporter Assays (MPRAs) (19,20) have emerged as important tools for investigations into the regulatory code, but these methods measure gene expression only, making it difficult to directly study the impact of TF binding on transcriptional regulation. Recent studies have performed ChIP-based binding measurements on libraries of promoter elements (21,22); however, these studies were unable to quantitatively measure binding energies or analyze cooperative interactions, features which are critical for dissecting TF function. To study the complex nature of TF binding in a quantitative manner and correlate this binding with gene expression, we have developed Calling Cards Reporter Arrays (CCRA), a novel tool that builds on the previously reported Calling Card method (23–25). CCRA measures TF binding and the transcriptional consequences of this binding for hundreds of synthetic DNA sequences in the yeast, *Saccharomyces cerevisiae*. We first demonstrate that CCRA measures TF binding at synthetic promoters and gene expression from a downstream reporter in a sensitive, accurate, and reproducible manner. We then apply CCRA to study TF–DNA interactions and show that the CCRA method is able to detect single nucleotide difference in the free energy of binding with a sensitivity that is comparable to *in vitro* methods. We then use CCRA to study how cooperativity dictates TFs binding *in vivo*, by analyzing the binding of the bHLH factor Cbf1p. We find that the cooperativity between Cbf1p dimers varies sinusoidally as the distance between two Cbf1p binding sites is changed, with an observed period of 10.65 bp. The helical phase of binding sites plays a major role in the cooperative binding of this factor, as ‘out of phase’ sites incur an energetic cost of 3.40 kJ/mol (1.37 *K*_B_*T*) relative to in-phase sites. Finally, we characterize the binding of a group of TFs that are thought to act together as a ‘TF collective’, a recently proposed model of cooperative binding (26,27). Consistent with previous work (23), we find that one member of the group, Tye7p, is able to bind at promoters that do not encode its recognition sequence. Surprisingly however, the binding of other collective members, Gcr1p and Gcr2p, requires only their recognition sites, suggesting a hierarchy where these factors can recruit Tye7p but not vice versa. We further demonstrate that the expression of a reporter gene regulated by this collective can be best explained by considering the occupancy of all members of this complex. Together, these results establish CCRA as a useful tool for quantitative investigations into TF binding and function.

## MATERIALS AND METHODS

### Library design and amplification

CCRA libraries are created by array-based oligonucleotide synthesis (Agilent). Each element of the library is a distinct 230 bp oligonucleotide comprised of five different sequence regions (See Supplemental Note 2 for a diagram of these regions and the specific sequences used in this study). The first region is a 20 bp constant sequence that is homologous to the backbone plasmid to support Gibson cloning. The next (downstream) 11 bp sequence is unique to each sub-library to enable the amplification of subsets of the library elements that are synthesized in each batch. This allows for the analysis of different TFs or the testing of different hypotheses using a single oligonucleotide synthesis. The third region is the 170 bp user-defined variable synthetic promoter sequence. This region is followed by 12 bp ‘promoter’ barcode that identifies the corresponding promoter sequence at Illumina sequencing step. Each promoter barcode is designed to be at least 3 bp different than all other barcodes to control for synthesis, PCR and sequencing errors. The last region of each library element is a constant 17 bp sequence used for PCR amplification. The library pool was synthesized by Agilent as 10 pmol of lyophilized nucleic acid (See Supplemental Note 2 for primer design and additional details for library amplification). To amplify the library, we used 0.15 ng of library DNA template in a final 50 μl PCR reaction. In each 50 μl reaction, we used 0.2 mM dNTP mix, 0.5 μM forward primer, 0.5 μM reverse primer, 1× Herculase II reaction buffer, 1 M Betaine, 0.15 ng DNA template in water, 1 μl of Herculase II polymerase (Agilent). The PCR reaction was cycled as follows: 95° for 1 min, 16 cycles of 95° for 30 s and 58° for 2.5 min and then 72° for 4 min. PCR products were purified by AMPure XP beads from Beckman coulter with 1:1.6 of PCR sample to magnetic particles ratio according to manufacturer's instructions. Typically, we obtained 5 to 10 ng/μl of DNA in a final volume of 15 μl.

### CCRA library construction

Plasmid pRS414 was used as the backbone to create library plasmid pRM1806 (See Supplemental notes 1a for plasmid map, sequence, and Supplemental Table S2 for Addgene accession number). To clone library sequences into the pRM1806 backbone, we linearized the plasmid with high fidelity KpnI and SacI (NEB), and then performed gel extraction using the Qiagen DNA extraction kit. We used 0.03 pmol of the linearized plasmid and 0.12 pmol of purified PCR product in a Gibson assembly reaction (NEB), following the manufacturer's instructions. Nitrocellulose membrane (0.025 μm) was used to filter Gibson assembly product by drop dialysis following the Millipore Sigma protocol. The library was electroporated into 10G SUPREME Electrocompetent cells (Lucigen) using 0.1 cm cuvette and cells were plated on to Kanamycin containing LB plates after 1-hour recovery in SOC. After 16 h of growth, over 50 000 colonies were scraped and the plasmid DNA was extracted using Qiagen Miniprep Kit.

### Calling cards induction and promoter library recovery

The yeast strain used in this study was yRM1004, which is derived from matA_deltaSir4, and has the following genotype: his3Δ1 leu2Δ0 met15Δ0 ura3Δ0 Δsir4::KanMx Δtrp1::HygMx. Induction of TF directed transposition was performed using a modified calling cards protocol (24). Briefly, plasmid containing a Sir4p (amino acids 951–1200) tagged TF driven by *ADH1* promoter with LEU2 auxotrophic marker was transformed into yeast cells (yRM1004) together with the plasmid pRM1804 (see Supplemental notes 1b for plasmid maps, sequences, and Supplemental Table S2 for Addgene accession numbers), which contains the URA3 marker and a galactose inducible Ty5 transposon with an artificial intron inside of His3 gene that is inside of Ty5 gene body for the purpose of selecting transposition positive cells in the next step (28). After transformation, cells were plated onto a Glu-Ura-Leu plate to select for cells carrying both the TF-sir4p fusion plasmid and Ty5 transposon plasmid. Next, a single colony was picked for library plasmid transformation. The library plasmid pRM1806 carries the TRP auxotrophic selection marker, so after the yeast cells were transformed with the library plasmid, they were plated onto a Glu-Ura-Leu-Trp plate to select for all three plasmids. Multiple parallel transformations were performed to obtain a diverse population of library sequences. We typically obtained over 10 000 colonies for each sub library. All colonies were pooled and plated to Gal-Ura-Leu-Trp to induce Ty5 transposition on 10 plates to increase the number of transpositions. Cells were allowed to grow on galactose plates for 4 days at room temperature. After galactose induction, we replica plated cells to Glu-His-Trp to select for yeast with Ty5 transpositions and that carry the library plasmid. After 2–3 days, colonies were scraped, and plasmid extraction was performed using the Yeast Plasmid Mini Kit (Omega).

### Preparation of Illumina libraries for calling cards mapping

We performed four independent PCRs to recover transpositions that were inserted into synthetic promoters in either of two possible orientations and upstream or downstream of the barcodes and UMI. We performed an additional PCR to measure the relative abundance of elements in the library for normalization. For these four PCRs, one primer of each pair is specific to either 3′ LTR of Ty5 transposon sequence or 5′ LTR of Ty5 transposon sequence, and the other primer is specific to a constant region either upstream or downstream of the inserted library sequence on the plasmid. For the additional PCR, one primer is specific to an upstream constant region of the inserted library sequence on the plasmid, and the other primer is for the downstream constant region (See Supplemental Table S1 for the primer sequences used). All five PCR products were pooled together for sequencing.

In each PCR reaction, we used 1X RedTaq buffer, 0.2 mM dNTP mix, 1M Betaine, 0.5 μM forward primer, 0.5 μM reverse primer, 4 μl RedTag DNA polymerase (Sigma-Aldrich), 1 μg of the purified plasmid DNA and the corresponding amount of water to reach a final volume of 50 μl. The PCR parameters were set to be 93° for 2 min, 24–28 cycles of 93° for 30 s and 62° for 6 min and 62° for 6 min. The PCR products were then purified with Qiagen PCR purification kit before sequencing.

### Measuring reporter expression in CCRA libraries by Sort-Seq

After transforming the library plasmid into yeast, we divided the cells for either Calling cards or Sort-Seq. For expression measurement, we followed the experimental procedures as well as promoter expression calculation described in (18). We sorted cells into eight bins of 100 000 cells each, and then added yeast culture media to grow the cells for 16 h. Cells from each bin were then pelleted separately and the plasmids were extracted with Yeast Plasmid Mini Kit (Omega) for sequencing.

Next, we performed a separate PCR reaction for each sorted bin. The primer sequences are listed in Supplemental Table S1, and they target the constant regions upstream and downstream of the CCRA library. In each of the eight PCR reactions, the reverse primer was indexed with unique barcode to allow the reactions to be sequenced together. The PCR amplification conditions used were identical to those used for calling cards recovery.

### Analysis of sequencing reads for quantification of TF binding

To quantify TF binding to CCRA libraries, we analyze Illumina paired end sequencing reads to count all unique insertions into each library member. An transposition is unique if it can be distinguished by its insertion coordinate relative to the library reference or contains a unique UMI in instances where multiple insertions have landed at the same position across four independent PCRs (See Supplemental notes 3 for examples of CCRA sequencing reads; our python analysis source code and an output sample are available on Gitlab). To identify unique insertions from the sequencing data, we first filter for reads containing the appropriate 12 bp library barcode and 6 bp TF barcode. Filtered reads are then divided into five categories: reads from synthetic promoters where the Ty5 transposon inserted in the forward direction upstream of the promoter barcodes, reads where Ty5 inserted in reverse direction upstream of barcodes, reads where the Ty5 inserted in forward direction downstream of barcodes, reads where the Ty5 inserted in reverse direction downstream of barcodes, and reads from synthetic promoters without insertion. This categorization is achieved by analyzing the first 20 bp of read 1 and read 2. The next 12 bp are used to map the precise location of the transposon insertion into the synthetic sequence. We used the 4 bp UMI to resolve events when multiple calling cards are deposited at the same base pair in a given synthetic sequence. Finally, we use the number of full-length sequences recovered for each library element as a normalization factor to control for the variation in abundance between library members. The total number of independent insertions for each library member is normalized by the relative abundance of each element in the library to compute a normalized binding score (NBS) of TF binding to each synthetic sequence.

### Using an expectation maximum algorithm to distinguish TF-directed insertions from background

For experiments in which changes in binding energies are measured, it is important to measure TF binding strength as accurately as possible. Therefore, we used an expectation maximization algorithm to resolve TF-directed transpositions which occur near TF recognition sites from background transpositions which occur uniformly across the synthetic promoter. Since the distribution of TF directed insertions is approximately Gaussian with the distribution centred at the TF recognition site, we assumed that TF directed insertions can be modelled with this distribution while background insertions follow a uniform distribution. We then used an expectation maximum algorithm to estimate, for each synthetic promoter, the variance of the Gaussian distribution (the mean value is determined by the location of the TF recognition sequence) and the fraction of insertions that were the result of a TF-directed or background transposition. For each library element, we iterate each independent insertion for maximum of 1000 times or until the parameters no longer change. The estimated fraction of TF-directed insertions is used to multiply the raw number of insertions at each promoter to remove insertions due to non-specific transposition (Python script and sample files are provided in Gitlab). This background correction step removes 0–20% of non-specific insertions, which is important for calculating small changes in binding energy; however, incorporating this step does not impact other analysis and should not be used for sequences where the Gaussian assumption is not appropriate (e.g. for sequences with multiple TF sites or for TFs whose recognition sequence is not well-characterized). Therefore, we performed this background correction only for the generation of binding energy landscapes.

### Binding energy difference calculation

To quantitatively compare CCRA with PBM and MITOMI in terms of binding affinity, we calculated the change of binding energy ($\Delta \Delta G$) from consensus site to the alternative site as follows:

Under binding equilibrium, [TF] and [sequence] associate at the same rate that the bound complex [TFS] disassociates:

(1) $$\begin{equation*}[{\rm{TF}}] + [{\rm{S}}] \longleftrightarrow [{\rm{TFS}}]\end{equation*}$$

The Gibbs free energy $\Delta G$ is related to the binding constant K as follows:

(2a) $$\begin{equation*}{{K({\rm S})}} = \frac{{[{\rm TF}][{\rm S}]}}{{[{\rm TFS}]}} = {{\rm e}^{\Delta G/RT}}\end{equation*}$$

(2b) $$\begin{equation*}\Delta {{G}} = {{RT\\ {\rm ln}}}({{K}}({\rm{S}}))\end{equation*}$$

The binding occupancy on a sequence is defined as the fraction of bound sequence to the total sequence in solution. Replace [TFS] with [TF][S]/K according to 2a, and by approximation that K(S) is much greater than [TF] as the affinity of these sequences are high, we get:

(3a) $$\begin{equation*}{\rm{Occ}}({\rm{S}}) = \frac{{[{\rm TFS}]}}{{[{\rm TFS}] + [{\rm S}]}} = \frac{{[{\rm TF}]}}{{[{\rm TF}] + K({\rm S})}} \approx \frac{{[{\rm TF}]}}{{K\left( {\rm S} \right)}}\end{equation*}$$

(3b) $$\begin{equation*}{\rm{K}}({\rm{S}}) = \frac{{[TF]}}{{Occ(S)}}\end{equation*}$$

Therefore, the change of binding energy equals:

(4) $$\begin{eqnarray*}\Delta \Delta G &=& \Delta G({\rm Sconsensus}) - \Delta G({\rm Smutant})\nonumber\\ &=& - {{RT{\rm ln}}}\left( {\frac{{{\rm Occ}({\rm Smutant})}}{{{\rm Occ}({\rm Sconsensus})}}} \right)\end{eqnarray*}$$

### Test for binding cooperativity

To determine if Cbf1p binds cooperativity at various synthetic promoters, we compared the observed occupancy to expected occupancy assuming independent binding, and we derived this test by the following:

$$\begin{eqnarray*}[{\rm{Cbf}}1{\rm{p}}] &+& [{\rm{DNA\,with\,two\,free\,sites}}]\overset {k1} \longleftrightarrow [{\rm{Cbf}}1{\rm{p}} - {\rm{DNA\,with\,one\,free\,site}}] \\ &+& [{\rm{Cbf}}1{\rm{p}}]\overset {k2} \longleftrightarrow [2*{\rm{Cbf}}1{\rm{p}} - {\rm{DNA\,with\,both\,sites\,occupied}}]\end{eqnarray*}$$

To simplify: $[{\rm{P}}] + [{\rm{S}}]\overset {k1} \longleftrightarrow [{\rm{PS}}] + [{\rm{M}}]\overset {k2} \longleftrightarrow \ [{{\rm{P}}_2}{\rm{S}}]$

$$\begin{equation*}{\rm{Occ}}({\rm{P}}) = \frac{{2*K1*P + 2*K1*K2*{P^2}}}{{1 + 2*K1*P + K1*K2*{P^2}}}\end{equation*}$$

If Cbf2 binds additively, then *K*1 = *K*2 = *K*;

$$\begin{eqnarray*}{\rm{Occ}}({\rm{P}}) &=& \frac{{2*K*P + 2*{k^2}*{P^2}}}{{1 + 2*K*P + {k^2}*{P^2}}} \\ &=& \frac{{2*K*P\left( {1 + K*P} \right)}}{{{{\left( {1 + K*P} \right)}^2}}}\\ &=& 2*\left( {\frac{{K*P}}{{1 + K*P}}} \right)\end{eqnarray*}$$

And so, the null expectation for binding occupancy is simple twice the observed binding to a single recognition site.

### TF motifs and NDS definition

For yeast TF motifs, we used the recommended PWMs compiled by Spivak and Stormo in the ScerTF database(stormo.wustl.edu/ScerTF). The ScerTF recommended PWM cutoff scores were used to define the presence or absence of TF sites on DNA sequences. The binding motif of MAX, the human bHLH factor, was obtained from factorbook (v1.factorbook.org/mediawiki/index.php/MAX). The NDS sequences used for this study were taken from a study by Raveh-Sadka (29); the NDS1 and NDS2 sequences in this work correspond to the v1 and v37 sequences from that study, respectively.

### Processing PBM and MITOMI data

Cbf1p PBM data was obtained from UniProbe database, and we used dataset UP00397 for calculating free energy changes. We searched for each motif variant in PBM data, all the sequences that contains the same motif variant are grouped together, and the average PBM score was used to reflect the binding affinity for that variant. MITOMI data was obtained from the study by Maerkl (30) and the *K*_d_ for each relevant variant reported in the original publication was used for the calculation directly.

## RESULTS

### Overview of Calling Cards Reporter Arrays (CCRA)

**Figure 1.**
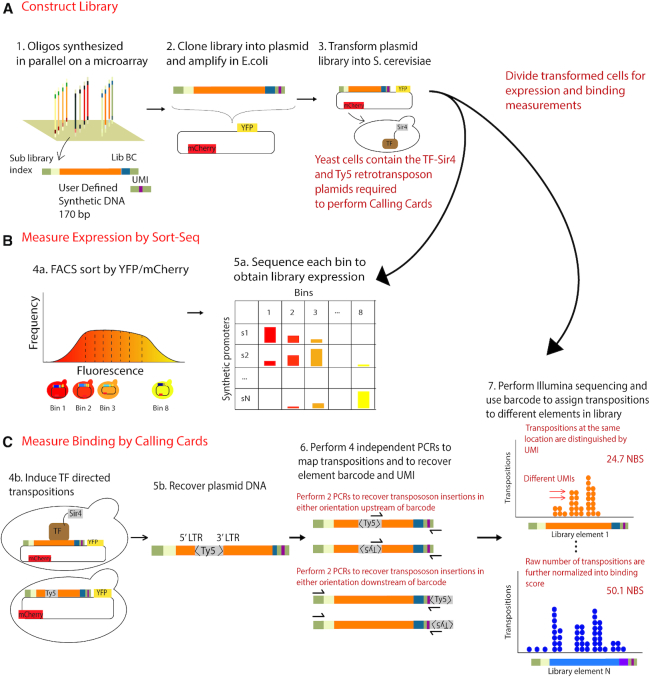
Illustrations of CCRA experimental steps and binding results recovery. (**A**) CCRA library sequences are synthesized on a microarray and cloned into plasmid and transformed into S. cerevisiae. The transformed cells are divided into two subpopulations for either binding measurements by Calling Cards method or expression measurements by Sort-Seq method. (**B**) Because the promoter library is cloned upstream of YFP reporter gene, and the mCherry reporter is constantly expressed from the same vector for internal control, cells are sorted based on the ratio of YFP and mCherry fluorescence to estimate the relative strength of the library promoter sequences. (**C**) Each element in the library is designed to contain a sub-library index that allows the user to assay a sub-population of the library, a unique barcode for identity, and a 4 bp randomized UMI to increase binding measurement capacity. TF-directed transpositions into barcoded library are fully recovered by four PCRs to account for insertions in either orientation and the relative position to barcode and UMI. PCR products are sequenced, and each library element is identified by barcode. Each dot represents a TF-directed transposition. The relative position of the insertion in the library sequence of each transposition is shown as X-axis. Multiple transpositions at the same position are distinguished by UMI. Raw number of transpositions are further normalized into a binding score (NBS) by correcting for the relative abundance of each element in the library as well as the total number of transpositions in one experiment to make accurate comparisons across experiments.

The CCRA method is designed to measure both TF binding and gene expression in parallel for hundreds of uniquely barcoded synthetic promoter sequences. To perform CCRA, the TF of interest is C-terminally fused to a short protein tag, so that the TF directs insertion of Ty5 retrotransposons (or ‘calling cards’) (24,25) near its binding sites (Figure 1A and C). For each CCRA assay, TF-directed insertions into the designed promoter library are recovered from yeast cells and the insertion locations and promoter sequence identities are determined via second-generation sequencing (Figure 1C). Each plasmid molecule in a CCRA library has a ‘library barcode’ corresponding to a unique promoter sequence (Figure 1A), as well as a unique molecular identifier (UMI). The library barcode allows each transposon calling card to be assigned to the correct synthetic promoter sequence, and the UMI enables us to determine when multiple transposition have inserted into the same location in distinct copies of the same synthetic promoter sequence. By determining the number of independent transpositions inserted into each synthetic promoter and then normalizing by the promoter's abundance in the library, we generate a normalized binding score (NBS), which is a quantitative measure of TF binding (Figure 1C).

Because the CCRA library is cloned upstream of a yellow fluorescence protein (YFP) reporter gene, it is also possible to measure the transcriptional output of each synthetic promoter in the library using Sort-Seq (17,18) (Figure 1B). To do so, the CCRA library is sorted by flow cytometry into subpopulations according to the ratio of YFP fluorescence to mCherry fluorescence. The mCherry gene is regulated by a constitutive promoter, allowing for normalization of the YFP signal to account for variation due to plasmid copy number, cell size, and other sources of extrinsic expression noise. Next, the sorted subpopulations of yeast cells are sequenced to quantify the abundance of each barcoded sequence in each subpopulation. Relative expression is then calculated by the proportion of each sequence in every binned library as per the standard Sort-Seq protocol (17,18). By combining aspects of both Calling Cards assay and Sort-Seq, CCRA allows us to quantitatively measure the binding of a TF to a library of regulatory sequences, and simultaneously measure the effect of that binding on gene expression.

### Binding and expression measurements are sensitive, accurate and reproducible

**Figure 2.**
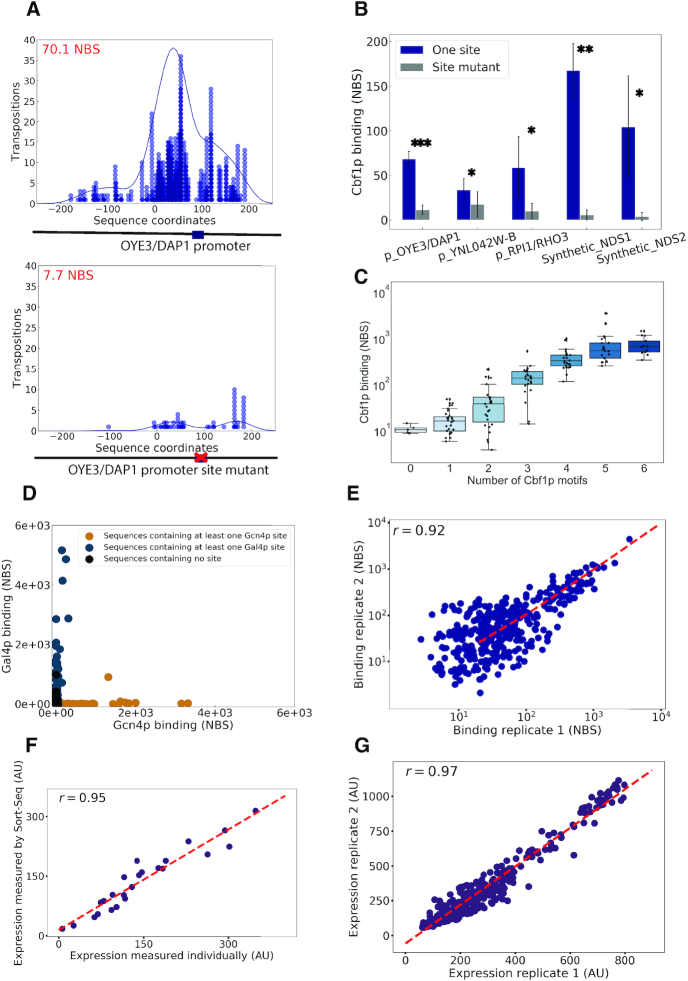
CCRA produce accurate and reproducible measurements on binding and expression. (**A**) Cbf1p directed transpositions into the *OYE3_DAP1* intergenic region where only one E-Box motif is present. Each dot represents a unique TF-directed transposition along the sequence. The x-axis specifies the sequence coordinate to which a calling card insertion was mapped, whereas the y-axis specifies the number of independent insertions at each position. Transpositions at the same position are distinguished by a UMI. In general, transpositions follow a gaussian distribution center at the transcription factor binding site. (**B**) Cbf1p binding measurements on three pairs of promoter regions and two pairs of synthetic sequences with one motif flanked by NDS. Blue bars represent sequences containing a motif, and gray bars represent the paired sequences with a mutated motif. The significance of binding detection on one motif is indicated by the number of stars. Three stars indicate a *P*-value of less than 0.0001 by paired *t*-test with four replicates, two stars, a *P*-value less than 0.001, and one star, a *P*-value <0.05. (**C**) Quantitative Cbf1p binding measurements on 183 sequences containing 0–6 motifs. Mean and standard deviation are indicated by the lines in each boxplot. The dynamic range spans over 3 orders of magnitudes. (**D**) Gcn4p and Gal4p were tested on a 344 elements library derived from Gcn4p or Gal4p naturally bound promoters. The library is categorized into three groups: sequences containing at least one Gcn4p site, sequences containing one Gal4p site and sequences containing no site. Most Gcn4p and Gal4p directed transpositions go to sequences containing at least one of either motif respectively, suggesting CCRA performs accurate binding measurement with little false positive. (**E**) Showing binding reproducibility from two binding experiments with Cbf1p on 531-element library. Pearson *r* = 0.92, *P*-value = 4.23e–216; Spearman *r* = 0.63, *P*-value = 3.21e–59. (**F**) 24 clones were measured by Flow cytometry individually and compared to the expression measured by Sort-Seq with Pearson correlation coefficient of 0.95. Pearson *P*-value = 6.59e–13; Spearman *r* = 0.96 and *P*-value = 7.91e–14. (**G**) Showing expression reproducibility on a 344-element library derived from Gcn4p and Gal4p binding targets. Pearson *r* = 0.97 and *P*-value = 1.51e–228, Spearman *r* = 0.95 and *P*-value = 1.29e–177.

To determine if CCRA can accurately and reproducibly measure TF binding in parallel, we first analyzed the binding of Cbf1p, a well-studied bHLH protein whose motif is strongly predictive of its *in vivo* binding pattern (23). To evaluate the sensitivity of the method for the detection of TF binding at weak sites, we created a library of 40 different sequences consisting of 10 synthetic promoters, each with four unique barcodes for replicates. Three of these sequences were taken from different endogenous yeast promoters previously shown to be bound by Cbf1p at a single recognition site (23). We also designed two synthetic promoters with nucleosome disfavouring sequences (29) that flanked a single Cbf1p consensus motif. As negative controls, we included five matched promoters with mutated Cbf1p binding sites. The binding of Cbf1p to a representative promoter, *OYE3/DAP1*, and its matched control is shown in Figure 2A. Each symbol on the graph represents an independent calling card insertion. Cbf1p-directed transpositions appear to fit a Gaussian distribution centred at Cbf1p motif. Interestingly, the region directly over the motif contains few insertions, likely due to Cbf1p's footprint as binds to its recognition sequence. The wild-type *OYE3/DAP1* promoter is bound tightly by Cbf1p (70.1 NBS), but when the Cbf1p binding site is mutated, binding is greatly reduced (7.7 NBS, Figure 2A bottom panel). Cbf1p's binding to all five pairs of promoters is summarized in Figure 2B. In all instances, Cbf1p's binding was significantly stronger at promoters with intact Cbf1p sites than at the mutated promoters, demonstrating that the CCRA method can reliably detect TF binding even at relatively weak sites containing single motifs. It is interesting to note that although Cbf1p binding was significant at all five promoters with intact Cbf1p motifs, the binding was significantly stronger at the two promoters in which the Cbf1p binding sites were flanked by nucleosome disfavouring sequences.

We next investigated the dynamic range of the CCRA assay. Since Cbf1p binding at regulatory elements is known to strongly depend on the number of Cbf1p sites present (23), we designed 183 synthetic promoters containing 0 to 6 sites and measured the binding of Cbf1p to this library. We observed a strong non-linear relationship between the normalized binding score (NBS), and the number of sites present in a given promoter (Figure 2C). Importantly, we were able to measure Cbf1p binding across 3 orders of magnitude. These data demonstrate that CCRA technology can accurately measure TF binding across a large range of binding strengths.

Because the oligonucleotides used to create the synthetic promoters for CCRA are typically 170bp in length, we next sought to determine if TFs still bind *in vivo* with the same specificity as they do in their native genomic context. Therefore, we designed a 344-element library of genomic promoters derived from endogenous Gcn4p and Gal4p target promoters and used CCRA to measure the binding of these two TFs. We found that Gcn4p directed transpositions almost exclusively to synthetic promoters derived from Gcn4p targets whereas Gal4p directed transpositions to Gal4p targets (Figure 2D), with each TF showing little non-specific binding to the other TF’s set of target sequences. These results indicate that truncated genomic sequences in a plasmid-based system still retain their specificities and are not aberrantly bound by other TFs.

Having established that the CCRA assay measures TF binding with high sensitivity and specificity, we next sought to benchmark the method's reproducibility. To do so, we performed replicate CCRA experiments using a 531-element synthetic promoter library and found that the NBS measured for each library member was highly reproducible (Pearson *r* = 0.92, *P*-value = 4.23e–216, Spearman *r* = 0.63, *P*-value = 3.21e–59. Figure 2E).

We next sought to establish that the CCRA method could accurately and reproducibly measure expression of the YFP reporter driven by a synthetic promoter library. To determine accuracy, we performed Sort-Seq to measure reporter expression for each member of a library containing sequences derived from Gcn4p and Gal4p promoters. We then cloned 24 of these library members and individually measured their expression levels by flow cytometry. We observed excellent agreement between the two measurements; the Pearson correlation coefficient was 0.95 (Pearson *P*-value = 6.59e–13, Spearman *r* = 0.96 and *P*-value = 7.91e–14), indicating that CCRA methodology accurately measures promoter activities from a library of synthetic sequences (Figure 2F). To further investigate the accuracy of the method using a functional approach, we evaluated reporter expression as a function of the number of TF recognition sites for Gcn4p in an amino acid starvation growth condition and for Gal4p in galactose (31–35). For both factors, reporter expression increased with the number of motifs, as expected from the known mechanism of action for these TFs (Supplementary Figure S2 ). Finally, we also showed that expression measurements are highly reproducible between two biological replicates (Pearson *r* = 0.97 and *P*-value = 1.51e–228, Spearman *r* = 0.95 and *P*-value = 1.29e–177 Figure 2G).

The CCRA assay requires that the TF of interest be fused to a fragment of the Sir4p protein. This can be achieved by tagging the TF at its endogenous locus or by expressing the fusion from a plasmid, which is more convenient for many experiments. To investigate whether TF fusions expressed from plasmids binds to CCRA libraries in a similar manner as TF fusions expressed at their endogenous loci, we measured the binding for each using the same 531 synthetic promoter library and observed a high concordance (*r* = 0.84, Supplementary Figure S1). We also confirmed that transcription factors tagged with the Sir4p fragment do not influence Sort-Seq expression measurements as they are highly correlated with measurements made using untagged proteins (*r* = 0.94 for Gal4p, *r* = 0.99 for Gcn4p, Supplemental Figure S3A and B). Tagging TFs with Sir4p also does not appear to affect their functions (Supplemental Figure S3C–F). Taken together, these results demonstrate that the CCRA method accurately and reproducibly measures the TF binding and expression consequences to a library of synthetic promoters

### Quantitative and high-throughput measurement of the binding energy landscapes of transcription factors *in vivo*

**Figure 3.**
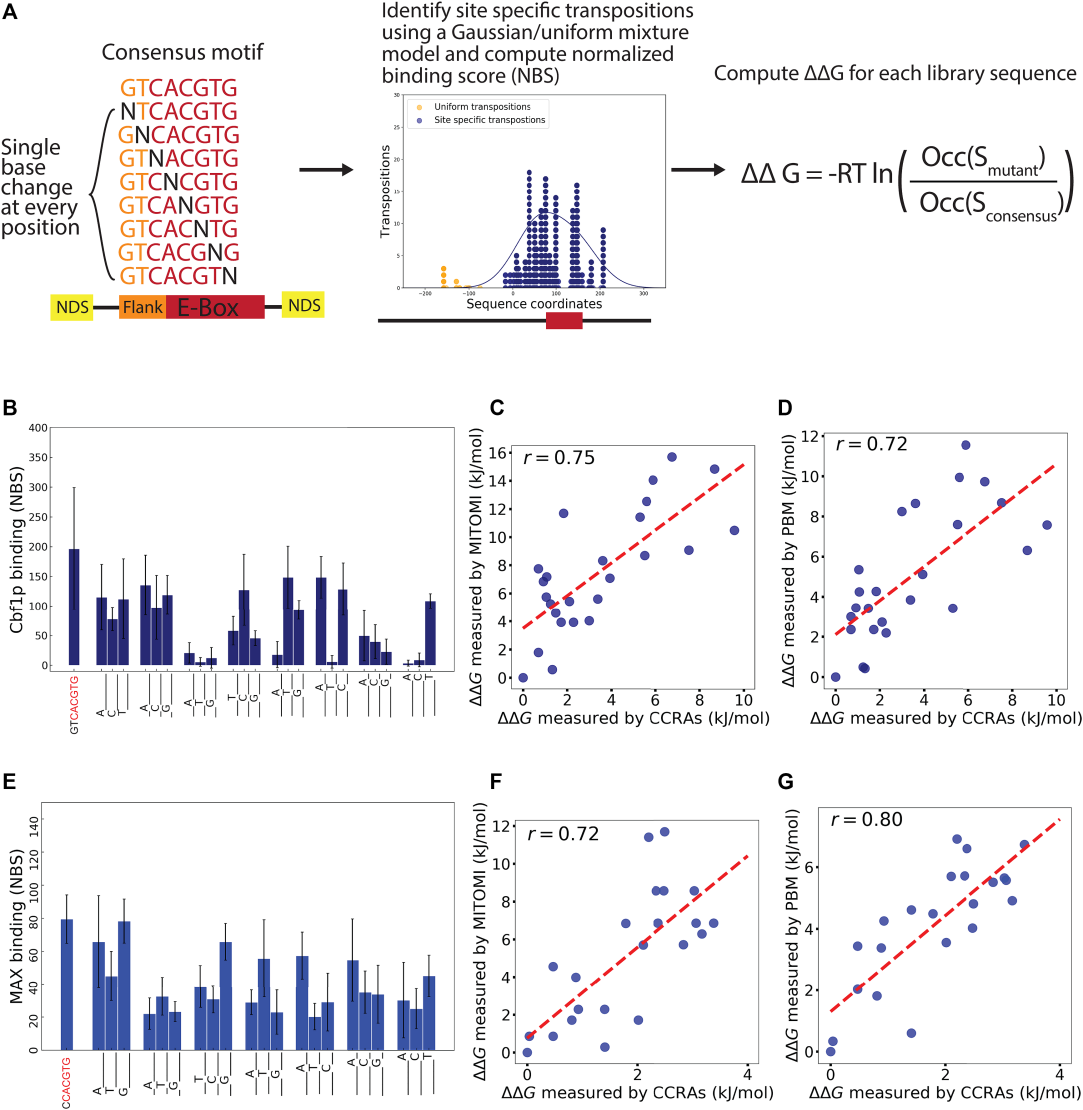
Binding energy measurements on alternative TF motif are quantitative. (**A**) Scheme of the experimental flow. A CCRA library was designed containing all possible alternative E-box motifs that are one base away from the consensus sequence and flanked with a nucleosome disfavoring site and analyzed for Cbf1p binding. Cbf1p directed transpositions were further processed using an expectation maximization algorithm (see Materials and Methods). The change of free energy was then calculated using the binding occupancy of the alternative motif and the consensus. (**B**) Cbf1p binding measurement on all alternative E-Box motif with four replicates. Standard deviation is indicated by the error bar. (**C**) The measured change of free energy for each alternative TF motif for Cbf1p was compared to the measurement by MITOMI. Pearson r of 0.75 and *P*-value = 1.90 e-5, Spearman *r* of 0.70 and *P*-value = 9.40e–5. (**D**) The same as (C) but compared to the measurement by PBM. Pearson *r* of 0.72 and *P*-value = 5.29e–5, Spearman *r* of 0.73 and *P*-value = 3.97e–5. (**E**) The same as (B) but with MAX transcription factor binding measurement. (**F**) The same as (C) but with MAX binding measurement. Pearson *r* of 0.72 and *P*-value = 1.42e–4, Spearman *r* of 0.74 and *P*-value = 9.06e–5. (**G**) The same as (D) but with MAX binding measurement. Pearson *r* of 0.80 and *P*-value = 7.24e–6, Spearman *r* of 0.77 and *P*-value = 2.36e–5.

Quantitative measurement of TF binding affinities to different DNA sequences is critical for understanding how TFs function *in vivo*. Because several studies have shown that minute variation in binding site affinity can specify alternative transcriptional or functional programs (36,37), it is important to be able to determine not only a TF’s consensus binding sequence, but also its binding energy landscape (i.e. the TF’s affinity for alternative binding sites). There are several methods that measure binding energy landscapes *in vitro*, such as MITOMI, PBM, Spec-seq, HT-SELEX, Bind-n-Seq, SPR, CSI and EMSA (30,38–49), and these have proven invaluable for understanding TF-DNA interactions. However, there is currently no method to accurately discriminate the small changes in free energy needed to generate binding energy landscapes *in vivo*. Such landscapes may differ from those measured *in vitro* due to the effects of nucleosomes and other chromatin-associated proteins on DNA shape and binding site accessibility. Therefore, we sought to determine whether CCRA could measure binding energy landscapes *in vivo*. We measured the binding of two basic helix loop helix (bHLH) factors, Cbf1p and MAX, to their consensus motifs and all sequences that differ by 1 bp from the consensus (Figure 3A). The TF binding sites were flanked by two intrinsic nucleosome disfavouring sequences to facilitate comparison to the *in vitro* binding landscapes previously determined (29). In order to accurately measure small changes in TF affinity, we used an expectation maximization algorithm to distinguish TF-directed transpositions from background insertions by assuming that TF-directed transpositions follow a Gaussian distribution centred at the consensus motif whereas non-specific transpositions follows a uniform distribution across the full synthetic promoter (see Materials and Methods). Cbf1p and MAX occupancies at their consensus binding sites and at all possible one base substitution are shown in Figure 3B and E respectively. As expected, both factors bound most strongly to their consensus sites. The changes in occupancies at non-consensus sites were strongly dependent on the position of the alteration and the identity of the substituted nucleotide. Some positions are crucial, such as the first position of core E-box motif, in the sense that any alternation resulted in completely abolished binding, whereas some positions such as flanking bases next to the core motif are more flexible when changed into other nucleotides. In general, Cbf1p binding appeared to be less tolerant to substitutions in its consensus motif than MAX, in agreement with previous *in vitro* measurements (30). We calculated the change of binding energy ($\Delta \Delta G$) from consensus site to the alternative site as follows (see Methods for a detailed derivation):

$$\begin{eqnarray*}\Delta \Delta G &=& \Delta G({\rm Sconsensus}) - \Delta G({\rm Smutant}) \\ &=& - {{RT{\rm ln}}}\left( {\frac{{{\rm Occ}({\rm Smutant})}}{{{\rm Occ}({\rm Sconsensus})}}} \right)\end{eqnarray*}$$

To determine whether the measurements performed by CCRA are concordant with the binding energy landscapes of Cbf1p and MAX as measured by well-established *in vitro* methods, we compared our results to MITOMI and PBM (Figure 3C, D, F and G). Both methods generated energy landscapes that were highly correlated to our CCRA measurements (For Cbf1p the correlation between CCRA and MITOMI: Pearson *r* of 0.75 and *P*-value = 1.90 e–5, Spearman *r* of 0.70 and *P*-value = 9.40e–5; the correlation between CCRA and PBM: Pearson *r* of 0.72 and *P*-value = 5.29e–5, Spearman *r* of 0.73 and *P*-value = 3.97e–5. For MAX the correlation between CCRA and MITOMI: Pearson *r* of 0.72 and *P*-value = 1.42e–4, Spearman *r* of 0.74 and *P*-value = 9.06e–5; the correlation between CCRA and PBM: Pearson *r* of 0.80 and *P*-value = 7.24e–6, Spearman *r* of 0.77 and *P*-value = 2.36e–5). Since the correlations between the measurements made by the two *in vitro* methods are similar in magnitude (for Cbf1p, the correlation between MITOMI and PBM: Pearson *r* = 0.73 and *P*-value of 3.35e–5, Spearman *r* = 0.78 and *P*-value = 4.57e–6. For MAX, the correlation between MITOMI and PBM: Pearson *r* of 0.79 and *P*-value = 1.28e–5, Spearman *r* = 0.79 and *P*-value = 1.25e–5 Supplementary Figure S4), these results demonstrate CCRA measures binding energy landscapes *in vivo* with an accuracy comparable to *in vitro* methods.

The reported binding constant (*K*) for Cbf1p and MAX is (6.2 ± 1.4) × 10^7^ M^−1^ at 20 °C (*K*_d_ = 1.6 nM) and (7.8 ± 2.6) × 10^6^ M^−1^ (*K*_d_ = 130 nM) respectively (50,51), and therefore the binding energy $\Delta G$ for Cbf1p is about –45 kJ/mol (−18 *K*_B_*T*) and –39 kJ/mol (–16 *K*_B_*T*) for MAX. Given the largest $\Delta \Delta G$ calculated from the consensus to the mutant motif, Cbf1p loses $\frac{1}{4}$ of its binding energy with one nucleotide difference (e.g. $\Delta \Delta G$ is 9.6 kJ/mol from GTCACGTG to GTCACGTA) and therefore the *K*_d_ on the mutated motif GTCACGTA becomes 71 nM, a 40-fold increase relative to the consensus motif. MAX loses $\frac{1}{{12}}$ of its binding energy with one nucleotide difference *in vivo* (e.g. $\Delta \Delta G$ is 3.4 kJ/mol from CACGTG to CACTTG), and therefore the *K*_d_ on the mutant motif is 500 nM.

### Quantitative measurement of the cooperative binding of Cbf1p

Understanding the mechanisms by which TFs select their targets *in vivo* will likely require more than just a characterization of their cognate DNA binding preferences, since it has been shown that many TFs achieve binding specificity through cooperative interactions with other DNA-binding proteins (10–15). Investigations into the cooperative interactions that occur between TFs are usually performed *in vitro*, under conditions that may not reflect the actual cellular environment (e.g. the lack of histones). *In vivo* investigations, which are less common, typically involve genome editing followed by quantitative binding measurement *in vivo*, which is experimentally challenging and time consuming (23,52,53). Given that CCRA is able to measure small changes in the free energy of TF binding, we sought to extend this approach to analyze TF–TF cooperativity. We focused on a pair of paralogous bHLH proteins, Cbf1p and Tye7p, both of which recognize the E-box motif CACGTG *in vitro* but bind to two distinct sets of target genes through different types of cooperative interactions.

**Figure 4.**
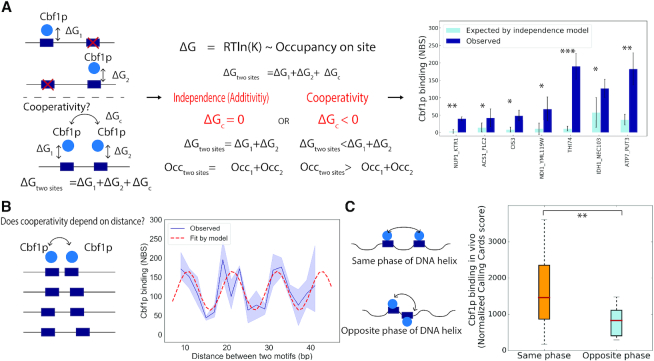
Cbf1p homotypic cooperativity is dependent on DNA structure. (**A**) Left and middle panel: experimental strategy to test if cooperativity exists between Cbf1p molecules when bind to sequences with multiple sites. Higher binding occupancy is expected than the sum of single site occupancy if cooperativity exists. Right Panel: Seven wild type promoters with either two or three motifs that are bound by Cbf1p were mutated such that only one motif was left. Binding on mutated sequences was combined and then compared to the binding on the wild type sequence to verify the existence of cooperativity between two Cbf1p molecules. The light blue bar represents the sum of the binding from individual motif, and the dark blue bar represents the observed binding on the wild type sequence. Error bar is the standard deviation across three biological replicates. One star indicates *P*-value less than 0.05, two stars indicate *P*-value <0.01 and three stars indicate *P*-value <0.001 by *t*-test. (**B**) Two Cbf1p motifs were positioned from 9 to 41 bp apart in 2 bp intervals. Cbf1p binding was measured with four replicates. A trigonometric function model was used to fit the observed data, and the period obtained was 10.65 bp. An ANOVA test was performed to assess the learned parameter with a *P*-value of 1.4e–6. (**C**) Genomic loci with two Cbf1p sites within 100 bp of each other were grouped according to whether they occur on the same side or opposite sides of the DNA helix (i.e. either separated by a multiple of 10.5 bp or by a multiple of 10.5 + ∼5 bp). Genomic Calling Cards score was compared between two groups, and a *t*-test was performed with *P*-value of 0.007.

We first set out to investigate Cbf1p, which has been shown to bind with homotypic cooperativity when two or more sites are present (23). This cooperativity was demonstrated by analyzing Cbf1p binding at mutated versions of the *IDH1_NCE103* divergent promoter, which normally contains three Cbf1p binding sites. This study showed that Cbf1p occupancy at the wild-type promoter was much stronger than the sum of the binding occupancies at three mutated promoters, each containing only a single Cbf1p binding site, demonstrating that Cbf1p binding is not additive but instead cooperative at this locus. However, in this study, Cbf1p's cooperativity was investigated at only a single promoter, so it is unclear to what extent this result can be generalized. We therefore sought to use CCRA to determine if this phenomenon occurs at other loci. We selected seven promoters with two or three Cbf1p sites including *IDH1_NCE103_pr* and designed a CCRA library in which these promoter sequences contained either zero, one or two mutated Cbf1p sites. If Cbf1p binds cooperatively at these loci, we expect that, for each series of synthetic promoters, the sum of the binding scores from sequences with a single Cbf1p site will be significantly less than the binding at the ‘wild type’ promoter sequence with multiple Cbf1p sites. In all seven cases, we found that Cbf1p binding at the wild type promoter was significantly higher than would be expected under an additive binding model (Figure 4A), suggesting that Cbf1p binds cooperatively at all target promoters that contain multiple recognition sites.

We next sought to characterize the relationship between the strength of Cbf1p cooperative binding and the distance between binding sites. Because the DNA double helix is thought to be rigid over length scale less than ∼140 bp due to vertical base-stacking interactions and intra-helix phosphate charge repulsion (54,55), one might expect that Cbf1p dimers would be unable to bind cooperatively at promoters with two recognition sites in close proximity. However, Cbf1p has been shown to sharply bend DNA upon binding (56–58), and, furthermore, DNA is clearly malleable to some proteins, as it is tightly wrapped around nucleosomes and can be twisted and untwisted during replication and transcription (59–61). To investigate the relationship between Cbf1p cooperativity and the distance between recognition sites, we designed synthetic promoters where we varied the distance between two Cbf1p consensus motifs from 9 to 41 bp with 2 bp intervals. We used CCRA to measure Cbf1p binding on these synthetic sequences, and plotted binding occupancy as a function of the distance between two sites. We found that the strength of Cbf1p binding at these synthetic promoters varied periodically with the distance between the binding sites (Figure 4B). We observed strong binding at the shortest distance of 11 bp, and we observed additional peaks at 22, 32 and 41 bp apart. These distances are all shorter than the persistence length of DNA, and at the longest distance investigated, 41 bp, the binding sites are separated by >65 Å, so it seems unlikely that the interaction between Cbf1p dimers could be explained by protein domain flexibility. Therefore, these results suggest that Cbf1p's ability to bend DNA allows the two dimers to interact with one another. We next hypothesized that the observed periodicity could be explained by the fact that Cbf1p makes its base pair contacts in the major groove of DNA so that at some motif distances, contact between Cbf1p dimers would require the rotation of the major groove around the axis of the double helix, incurring an energetic penalty. To test this, we fitted the binding to a cosine function. The calculated period was 10.65 bp, almost exactly the number of base pairs required for DNA to make one complete helical turn about its axis. We evaluated the fit of this model using analysis of variance (ANOVA) and obtained a *P*-value 1.4e–6, indicating that the data follows the assumed model significantly better than expected by chance. This result suggested to us Cbf1p dimers that are not bound on the same side of the DNA helix must twist the DNA and incur an energetic cost. In contrast, two Cbf1p molecules on the same face of the helix are able to achieve the optimal cooperative binding efficiency. We next sought to compute the free energy cost associated with twisting the DNA double helix. Since we observed a 3.8-fold difference between the highest and the lowest occupancy, we calculated that the free energy lost due to twisting is 3.40 kJ/mol (1.37 *K*_B_*T*). Compared to $\Delta \Delta Gs$ calculated for the consensus to mutant motif from the previous section, the energic cost of DNA twisting is comparably to a mild nucleotide change in the E-box motif (e.g. from GTCACGTG to GTCTCGTG). Interestingly, over the distance range examined in this experiment, the amplitude of the periodic function did not change appreciably, suggesting that, in contrast to twisting, Cbf1p bends DNA efficiently, with little energetic cost.

We next asked if the phase of Cbf1p binding sites influenced the binding of this transcription factor at native genomic loci. We took published genome-wide Cbf1p Calling Cards data (23) and grouped all intergenic regions with two Cbf1p binding sites within 100 bp according to the relative phase of the two sites. We found that promoters containing two Cbf1p binding sites separated by a multiple of 10.5 bp (i.e. with major grooves on the same side of the DNA helix) were bound significantly more tightly by Cbf1p than promoters with binding sites whose major grooves were on opposite sides of the DNA helix (Figure 4C, *P* = 0.007). This result demonstrates that the periodicity in cooperative binding that we observed in our CCRA experiments also influences Cbf1p binding in the yeast genome.

### The binding logic of the Tye7p/Gcr1p/Gcr2p/Rap1p TF collective

Unlike Cbf1p, many of the promoters bound by Tye7p do not encode an E-box, this factor's preferred binding motif (23). It has previously been shown that Tye7p binds cooperatively with the Gcr1p/Gcr2p/Rap1p complex and that by taking into account the DNA binding preferences of these proteins, the *in vivo* binding of Tye7p can be more accurately predicted (23). However, the biophysical principles that govern the binding of this complex are still unclear. For example, the binding of this complex does not appear to follow either of the two most well-studied models for TF binding, the Enhancesome model or the Billboard model (62,63), because these models both posit a one-to-one correspondence between the binding of a TF and the presence of its recognition site. Instead, Tye7p binding appears to be consistent with the recently described TF collective model, in which a group of TFs bind together, but the motif positioning and composition at target sites is flexible (26,27). However, the TF collective model is ambiguous with regard to the mechanistic details of binding, so important questions about the function of the Tye7p/Rap1p/Gcr1p/Gcr2p collective remain.

**Figure 5.**
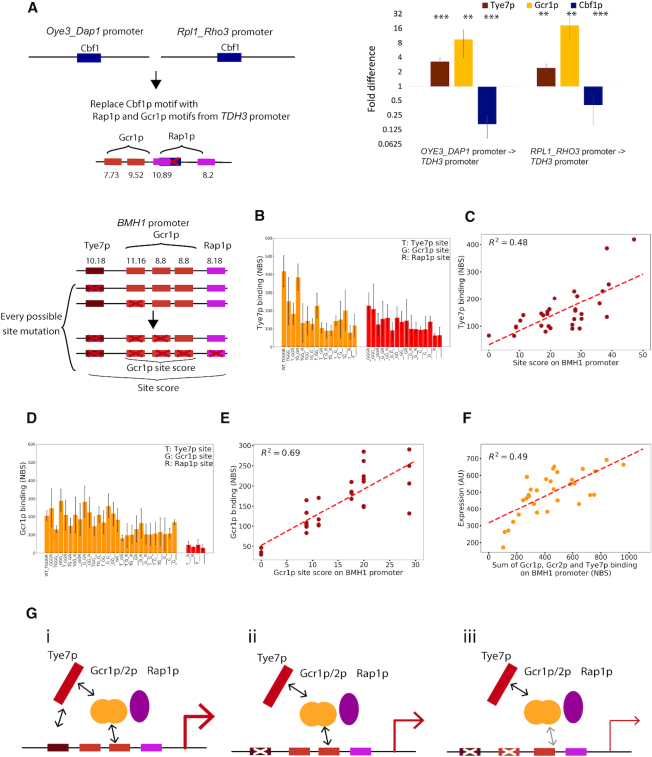
The molecular logic of Tye7p binding collective. (**A**) To test if Tye7p is able to bind without its motif through protein-protein interactions with Gcr1/2p and Rap1p, the Cbf1p motifs on the *Oye3_Dap1* and *Rpl1_Rho3* intergenic regions were mutated and two Gcr1p and two Rap1p motifs from *TDH3* promoter were added. Binding measurements were performed on the wild type and reprogrammed sequences for Tye7p, Gcr1p and Cbf1p. Tye7p bound to both reprogrammed promoters at significantly higher levels than the wild type *Oye3_Dap1* and *Rpl1_Rho3* sequences, as did Gcr1p. Cbf1p binding was abolished on these regions after mutation. T test was performed to assess the significance, and two stars indicate *P*-value <0.01 and three stars indicate *P*-value <0.001. (**B**) The *BMH1* promoter, bound by Tye7p, contains one Tye7p motif, three Gcr1/2p motif and one Rap1p motif; a CCRA library was created in which all combinations of sites were mutated to create 32 sequences, including the wild-type sequence. Tye7p binding was measured on these sequences and plotted. Intact sites are indicated as the x-axis label. All 32 sequences were classified into two sections, those with and without the Tye7p motif. Error bars represents the variation between four biological replicates. (**C**) The total binding free energy on each sequence based on the PWM score of the remaining sites was correlated with Tye7p binding result, and the total free energy of binding to DNA for the binding collective predicts Tye7p binding with *R*^2^ = 0.48, Pearson *r* = 0.69 and *P*-value = 1.07e-5, Spearman *r* = 0.63 and *P*-value = 1.04e–4. (**D**) The same as (B) but with Gcr1p, and these 32 sequences are classified into with and without any Gcr1/2p motif. (**E**) PWM score of Gcr1/2p sites remained on the sequences was correlated with Gcr1p binding result, and Gcr1/2p sites alone predicts Gcr1p binding with *R*^2^ of 0.69, Pearson *r* = 0.83 and *P*-value = 3.15e-9, Spearman *r* = 0.83 and *P*-value = 3.59e-9. (**F**) Expression was measured for all mutated sequences derived from *BMH1* promoter and was correlated with the summation of Gcr1/2p and Tye7p binding results. The binding of three factors from the collective predicts the expression with *R*^2^ of 0.49, Pearson *r* = 0.70 and *P*-value = 8.34e–6, Spearman *r* = 0.67 and Spearman *P*-value = 2.95e–5. (**G**) The suggested model for Tye7p/Gcr1p/Gcr2p/Rap1p binding collective. (i) Tye7p is recruited to promoters by Gcr1/2p and the Tye7p motif, and the expression output is the strongest when all sites are available; (ii) Tye7p can be recruited in the absence of a Tye7p motif via a protein-protein interaction with Gcr1/2p, but Tye7p binding occupancy is lowered and the overall expression output is lowered as well; (iii) Gcr1/2p occupancy and Tye7p occupancy are lowered with fewer Gcr1/2p motifs, and the overall expression output is further reduced.

We first assessed the predictive power of the collective model by attempting to reprogram yeast promoters that normally bind Cbf1p, a Tye7p paralog, into promoters that bind Tye7p. To do so, we took two promoters, *OYE3_DAP1_pr* and *RPL1_RHO3_pr*, that are normally bound by Cbf1p, and removed their E-boxes (i.e. Cbf1p/Tye7p binding sites), and added Gcr1/2p and Rap1p sites with a design based on the *TDH3* promoter, which is bound by Tye7p. We then assessed the binding of Tye7p to these reprogrammed promoters using CCRA. Both showed significant decreases in Cbf1p binding (6.1-fold and 2.4-fold respectively) and significant increases in Tye7p (3.3-fold and 2.4-fold respectively) (Figure 5A). We also observed an increase in Gcr1p binding at these reprogrammed promoters. Since neither of these reprogrammed promoters contain a consensus Tye7p binding site, we conclude that Tye7p binding is consistent with the collective model and that this TF can be recruited to promoters via cooperative interactions with Gcr1/2p and Rap1p.

Next, we wanted to better understand the molecular logic by which this collective binds. While Tye7p clearly does not require its motif to be present at a regulatory target, is this true for other members of the collective? When more than one binding site is present for a single TF, do the additional sites contribute to complex stability, or is one site sufficient and the others redundant? How is transcriptional output correlated with binding of each TF member? To answer these questions, we took a Tye7p bound promoter, *BMH1*_*pr*, which contains one Tye7p site, three Gcr1/2p sites and one Rap1p site, made every possible combination of mutated sites, and measured Tye7p binding using CCRA. Since Tye7 does not require its recognition sequence for binding, we first wanted to know if its motif made any energetic contribution to stabilize this factor. We divided the mutated sequences into two categories, those with and without a Tye7p motif. Sequences without a recognition site were still significantly bound by Tye7p (Figure 5B, right group), consistent with previous observations, but Tye7p binding at the wild-type *BMH1*_*pr* is reduced by 45% when the Tye7p recognition site is mutated (*P*-value = 0.012). Furthermore, when the 16 pairs of *BMH1_pr* mutants are compared across groups, we observe a significant reduction in Tye7p when the recognition motif is mutated (*P*-value = 0.010). These results demonstrate that while the Tye7p motif is not required for Tye7p binding, it makes an energetic contribution when present. Notably, the positional distributions of Tye7p insertions across the *BMH1_pr* were essentially unaffected by the presence or absence of its cognate motif (Supplementary Figure S6), suggesting that the recruitment of Tye7p may be largely mediated by Gcr1/2p and Rap1p, even though the presence of a Tye7p binding site clearly makes an energetic contribution. Consistent with this hypothesis, we found that Tye7p binding is strongly dependent on Gcr1/2p and Rap1p sites (Figure 5B). In general, we observed a gradual decrease in binding as more collective sites are mutated, and we did not observe large decrease in binding (>2 fold) upon the removal of any one site, suggesting that no single binding site is necessary for Tye7p binding at this promoter, but instead that all sites contribute to the binding affinity of this TF. Based on this observation, we reasoned that Tye7p binding might be predicted by the total free energy from all sites combined on a promoter. Therefore, we performed a regression analysis to understand how well the total sites information explains Tye7p binding (Figure 5C). Given that PWM scores reflect the binding energy of TF to specific DNA sequences, we used the sum of PWM scores for all sites present on the promoters for the analysis and we found that the combined sites information correlates well with Tye7p binding (Pearson *r* = 0.69 and *P*-value = 1.07e–5, Spearman *r* = 0.63 and *P*-value = 1.04e–4).

We then measured Gcr1p and Gcr2p occupancy on this promoter library. As before, we divided the mutated promoters into two categories based on whether they contained a Gcr1/2p motif. In contrast to what was observed for Tye7p, we found that neither Gcr1p nor Gcr2p was able to bind at any promoters without their shared recognition site (Figures 5D & Supplementary Figure S7A), suggesting that these factors bind independently from the rest of the collective. To confirm this, we regressed Gcr1p and Gcr1p binding against the free energy of binding of Gcr1/2p or the full collective. We found that only Gcr1p/2p sites are required to explain Gcr1p and Gcr2p binding and that incorporating information from the other TF in the collective weakens the predictive power (Figure 5E & Supplementary Figure S7D for Gcr1p and Supplementary Figure S7B & CS for Gcr2p). Thus, the binding of the Gcr1/2p complex appears to be solely dependent on the presence and the number of Gcr1/2p sites. Furthermore, Gcr1/2p binding appears to saturate at two sites. Our Gcr2p binding measurements were more variable and weaker than our Gcr1p measurement, especially at sequences with only one Gcr1/2p motif, which might be due to the fact that Gcr2p is known to bind DNA indirectly through Gcr1p and depends on Gcr1p to function (64,65).

We next sought to investigate the relationship between the binding of the Tye7p collective and its transcriptional output. To do so, we performed Sort-Seq to measure the reporter gene expression from this library. We regressed reporter gene expression against the sum of the free energies of the binding sites (Supplementary Figure S7E). We observed a good correlation, and we found that expression level correlated with the combined TF occupancy (Figure 5F, Pearson *r* = 0.70 and *P*-value = 8.34e–6, Spearman *r* = 0.67 and *P*-value = 2.95e–9), suggesting that transcriptional output is determined by the whole complex. Similar analysis was done for *TDH3* promoter containing two Gcr1/2p sites and two Rap1p sites but no Tye7p site, and again the combined Tye7p, Gcr1p and Gcr2p occupancy correlated well with the expression (Supplementary Figure S7F & GS).

Rap1p binding was not measured in this study due to its inability to be tagged by Sir4p. However, Rap1p has been shown to interact with Gcr1p and Gcr2p as an activating complex (66,67). With expression we measured on both *BMH1* and *TDH3* promoters, we compared sequence pairs that are with and without Rap1p site (Supplementary Figure S7H). We performed a paired T-test on these sequence in terms of expression, and the *P*-value is 0.018, indicating Rap1p motif is contributing the genetic regulation.

Taken together, our experiments suggest that Tye7p is recruited to promoters by Gcr1p/Gcr2p/Rap1p complex and that Tye7p binding often occurs in the absence of its recognition site. However, it appears that Tye7p binding is stabilized by the presence of its motif. In contrast, the Gcr1/2p recognition site is necessary and sufficient for the binding of these proteins, suggesting a hierarchy in which these factors can recruit Tye7p but not vice versa (Figure 5G). The transcriptional output at promoters bound by this complex correlates with the combined occupancy of all TFs, suggesting that each TF in the collective aides in the recruitment of the RNA Polymerase II holoenzyme.

## DISCUSSION

In this study, we demonstrated that the CCRA method is a useful tool to study many different aspects of TF binding *in vivo*. Using CCRA, we first measured the DNA binding energy landscapes for Cbf1p and MAX, and we showed that the free energy differences measured by CCRA are strongly correlated with those measured by PBM and MITOMI, suggesting CCRA is a quantitative measure of equilibrium binding. This is likely because the rate of transposon insertion is slow relative to the typical on rates and off rates for TF binding to DNA; in contrast, crosslinking based methods may capture transient TF-DNA binding events as TFs sample weak binding sites (68), and thus the measured occupancies may reflect a combination of on-rate and equilibrium binding. Next, we set out to understand TF cooperativity by studying a pair of paralogues bHLH TFs, Cbf1p and Tye7p; we observed that Cbf1p binding occupancy is dependent on the DNA helix turn, revealing the biophysical relations between DNA structure and a homotypic cooperative TF; Finally, we characterized the molecular binding logic of Tye7p, which is Tye7p finds its targets via protein-protein interaction with Gcr1/2p and Rap1p without requiring its own motif, further delineating the collective binding model.

Transcription factors orchestrate the gene expression changes that lie at the heart of most biological processes; however, the principles by which TFs locate their target genes and the functional consequences of binding are not well understood. Detailed investigations into the molecular mechanisms that govern TF binding have traditionally used *in vitro* methods (30,38–49), which provide limited insights into TF binding *in vivo*, or employ genome editing (23,52,53), which is slow and costly. Due to these difficulties, many studies that have tried to understand the rules of TFs binding and function have focused on a finite set of loci and a limited number of genetic alternations (23,52,53). Recently, powerful high-throughput methods, such as Sort-Seq (17,18) and barcoded MPRAs (19,20), have been developed to allow more comprehensive investigations into the regulatory code, but these rely solely on reporter gene expression and must indirectly infer TF binding and its impact on gene expression. Two recent studies have coupled ChIP-based binding measurement with parallel reporter assays to reveal the correlations between chromatin marks and TF binding (21) and to examine the predictive power of thermodynamically motivated models of gene expression (22). These studies demonstrated the parallel measurement of TF binding on synthetic promoters and represent an important advance; however, neither demonstrated the ability to quantitatively measure binding energies or to analyze cooperative interactions, which are critical measurements for understanding how TFs function. Methods in which TFs direct transposon insertion (24,25,69) or the enzymatic cleavage of DNA (70,71) show promise for going beyond a qualitative description of TF binding. Here, we demonstrate that CCRA is able to quantitatively measure TF binding and reporter gene expression on synthetic sequences in a high-throughput manner. It is a sensitive and accurate method that is amenable to the analysis of complexes of TFs. Therefore, CCRA should be a useful tool to better understand the regulatory principles of TFs localization and functionality.

When designing a CCRA library, certain considerations should be accounted for in order to ensure the accurate quantification of TF binding. It is important to collect enough transpositions events in each experiment relative to the size of the CCRA library. Although chip-based oligonucleotide synthesis allows for very large libraries (up to 244 000 unique oligos) to be synthesized in a cost-effective manner, we have found that it is advantageous to design the library so that smaller subsets (e.g. 100–1000 sequences) can be amplified with unique primer pairs. Since we typically collect 10 000–50 000 transpositions for each CCRA experiment (using 10 yeast plates), limiting the sub-libraries to this size ensures high statistical power for each experiment, while still allowing for the analysis of different TFs or the testing of different hypotheses in a single experiment. The optimal number of transpositions for a particular CCRA experiment will also depend on the transcription factors to be analyzed and the specifics of the library design (e.g. a library consisting of many high affinity sequences may yield more transpositions than library consisting of many low affinity sequences). In our experience, CCRA libraries with 500 or fewer unique sequences yield high-quality binding results, but this could be easily scaled by using more plates or through future improvements to the method. In the future, it should be possible to analyze multiple TFs simultaneously with CCRA technology by adding different TF barcodes during the first amplifying step and then transforming the barcoded libraries into different yeast strains, each containing a different TF-Sir4p fragment fusion.

The CCRA method is able to analyze a number of user-defined sequences in parallel, providing quantitative and well-controlled measurements that would be difficult to obtain using genome-wide methods. For example, the free energy binding landscape we described for Cbf1p was generated by analyzing all 1bp substitutions to this factor's consensus motif in exactly the same sequence context, a design which enabled the detection of small free energy changes. In contrast, small changes in binding energy cannot be inferred from genome-wide calling card measurements of Cbf1 (Supplementary Figure S5), although the broad trends are generally the same. This is likely due to the fact that while all 1bp substitutions to Cbf1p's consensus binding sequence are indeed present in the genome, they exist in different local sequence contexts, so the measurements are not well controlled. For example, in the yeast genome, one Cbf1p binding site might compete with a nucleosome, while another binding site may not, so the different local contexts confound the accurate measurement of binding energies. Indeed, we observed in our CCRA experiments that when a Cbf1p binding site is flanked with a nucleosome disfavouring sequence, Cbf1p binding consistently increases (Figure 2B). The ability to make well-controlled measurements likely also contributed to our ability to detect the periodic phase dependence of Cbf1p's cooperativity. This phase dependence is an interesting phenomenon, and to our knowledge cooperative binding of a transcription factor complex has not been previously shown to be influenced by helical phase. However, an important related result was found by Kosuri and colleagues where they found that the expression output of a reporter gene depended on the helical phase between the transcription start site and the binding site of a transcriptional activator (72).

We envision CCRA will be broadly applied to study three different aspects of TF binding: (i) quantitative investigations into TF–DNA interactions in the native cellular environment; for example, mapping TF binding energy landscapes *in vivo* or evaluating the effect of flanking sequences on motif recognition; (ii) studies into the mechanisms by which TFs bind cooperatively; for example, evaluating the energetic contributions of different TF binding sites to the binding of a TF complex; (iii) dissection of the relationship between TF occupancy and transcriptional output. Furthermore, it is likely that CCRA can be extended to multicellular eukaryotic systems in the future using the appropriate transposon machinery. The Calling Card method has been applied to study mammalian TFs such as SP1 and BAP1 with PiggyBac transposon (73,74), so this transposon system is an excellent candidate for performing CCRA in mammalian cells. Such investigations should ultimately lead to a better understanding of the roles that TFs play in orchestrating the transcriptional networks that allow cells to carry out their diverse functions.

## DATA AND SCRIPT AVAILABILITY

Synthetic DNA library and the analyzed results are provided as a excel spreadsheet. Scripts and samples for analysis of sequencing reads for TF binding quantification and expectation maximum algorithm for filtering are provided in https://gitlab.com/JiayueLiu/ccra_codes.git. Raw sequencing reads are available in GEO with series number GSE144437.

## Supplementary Material

gkaa141_Supplemental_Files

## Notes

Present address: Robi D. Mitra, Alvin Goldfarb Professor of Computational Biology, Department of Genetics, Washington University School of Medicine in St. Louis, St. Louis, MO 63108, USA.
