# Supplementary material for: Quantitative analysis of transcription factor binding and expression using calling cards reporter arrays
Source: Nucleic Acids Res. 2020 Mar 5;48(9):e50. doi: 10.1093/nar/gkaa141 (PMC7229839; doi:10.1093/nar/gkaa141)
Supplement: gkaa141_Supplemental_Files [file gkaa141_supplemental_files.zip › Supplemental_Info_CCRA_final_.pdf]

Quantitative Analysis of Transcription Factor Binding and Expression Using Calling Cards Reporter Arrays

SUPPLEMENTAL FIGURES

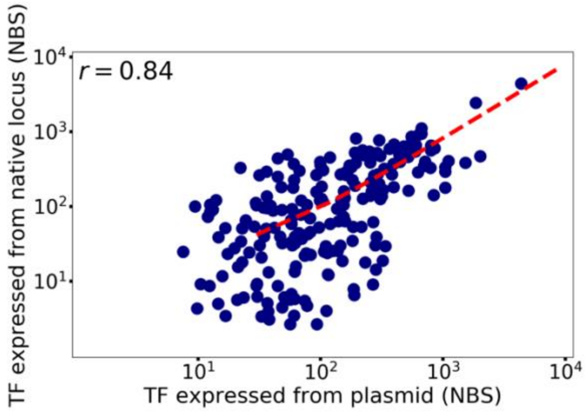

Figure 1S. Binding of Cbf1p-Sir4 expressed from a plasmid is well-correlated with binding of Cbf1p-Sir4 expressed from the native locus. The CCRA library used here was identical to the one used in **Figure 2E** .

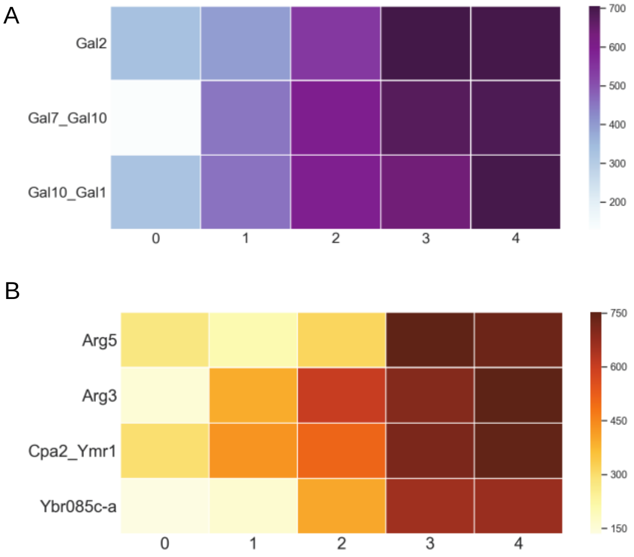

Figure 2S. Expression measurements were performed for a library consisting of synthetic promoters derived from Gal4p- and Gcn4p-regulated promoters. The number of corresponding motifs for each TF was varied in the library. **A)** reporter gene expression increases as the number of Gal4p motifs increases under galactose condition. **B)** reporter gene expression increases as the number of Gcn4p motifs increases under amino acid starvation condition.

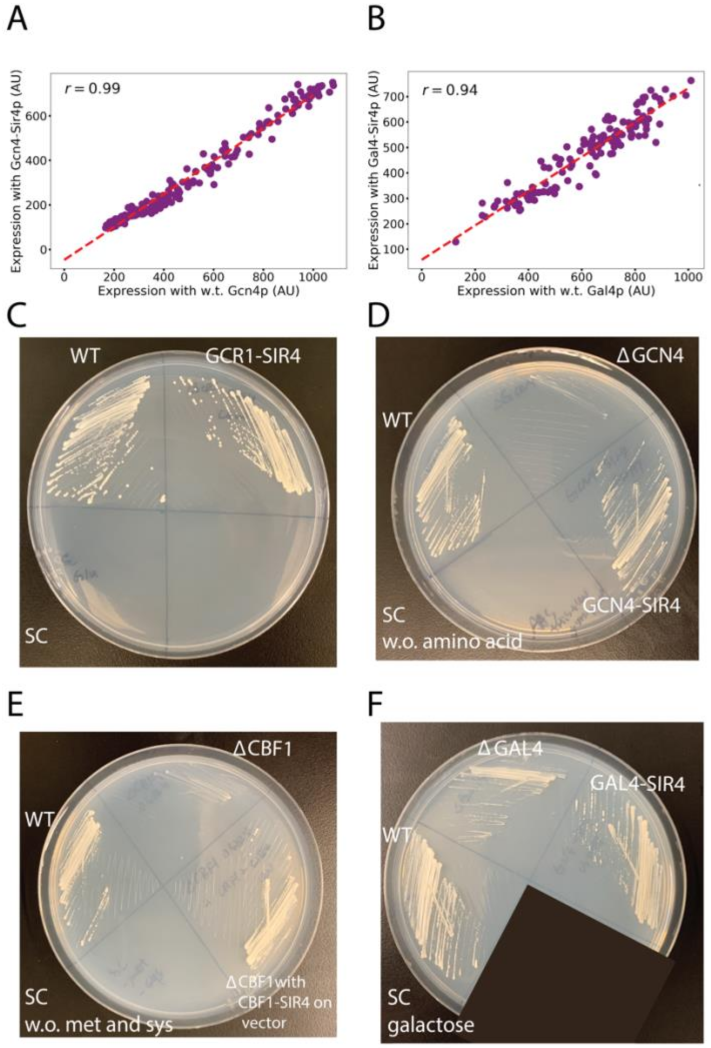

Figure 3S Comparison of Sir4p tagged and untagged transcription factors. To determine if Sir4p tagged TFs produce the same Sort-Seq measurements of gene expression, we took the Gcn4p and Gal4p CCRA library and performed Sort-Seq in an untagged w.t. background and compared the results to those obtained with the tagged TFs. Expression measurements for wild-type and Sir4p-tagged **A)** Gcn4p, and B) Gal4p were highly correlated; To determine whether the Sir4p affects TF function, we analyzed four different Sir4p-tagged TFs to see if they could rescue growth in a deletion strain grown under conditions where the TF is required. **C)** Gcr1p tagged with Sir4p is viable in yeast grown in SC; **D)** Gcn4p tagged with Sir4p is viable under amino acid starvation condition; **E)** Cbf1p tagged with Sir4p expressed from plasmid can rescue Cbf1p deletion strain under MET and CYS deficient condition; **F)** Gal4p tagged with Sir4p recovers the normal growth of yeast under galactose condition.

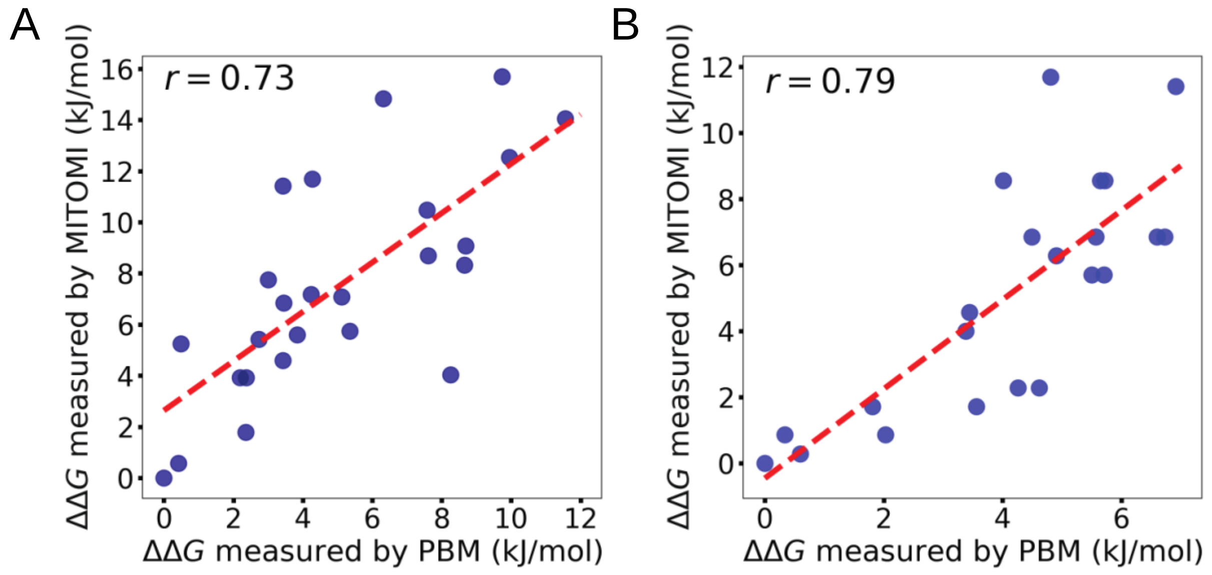

Figure 4S. A) Comparison of the change of binding energy measured by PBM and MITOMI for Cbf1p. **B)** The same as panel A, but with MAX transcription factor.

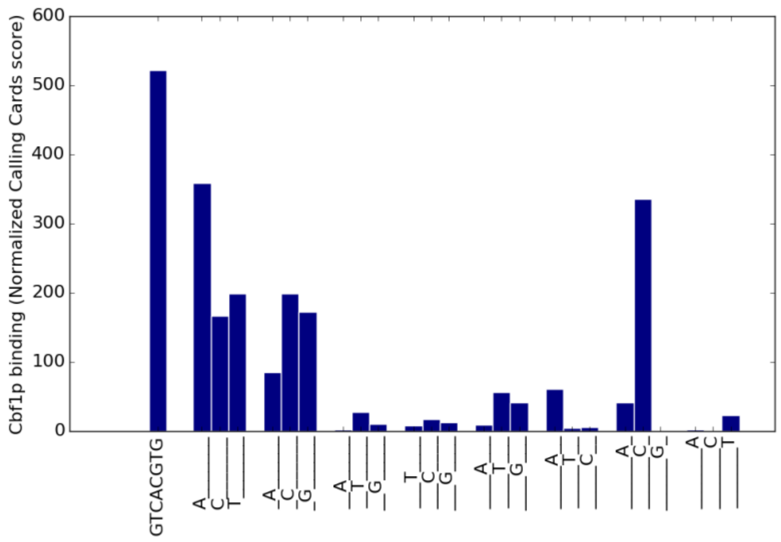

Figure 5S. Average *in vivo* genomic Cbf1p Calling Cards binding score on all alternative E-box motif. As expected from our CCRA binding energy landscape, mutations to the core CACGTG had a larger impact on Cbf1p binding than non-core motifs, but the effect was exacerbated in vivo, perhaps because of competition with nucleosomes. It is important to note that it would be impossible to generate accurate Cbf1p binding energies (benchmarked against *in vitro* measurements) solely from the *in vivo* binding data. This is because in the yeast genome, Cbf1p binding sites (and 1bp mutant sites) occur in a variety of different sequence contexts, whereas in the CCRA experiments, the Cbf1p sites were analyzed in precisely the same sequence context.

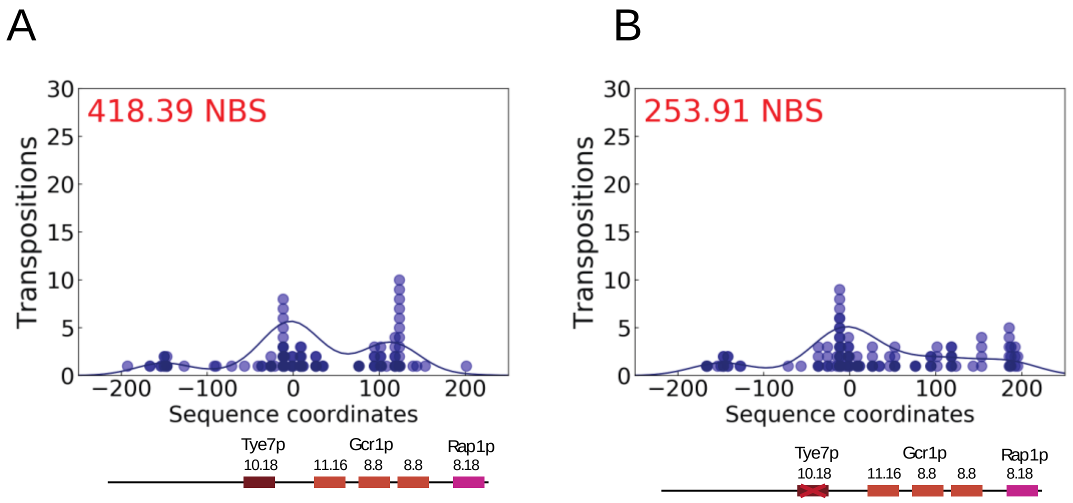

Figure 6S. Tye7p transposition distribution on **A)** w.t. *BHM1_pr* promoter and **B)** Tye7p motif mutated *BHM1_pr* promoter.

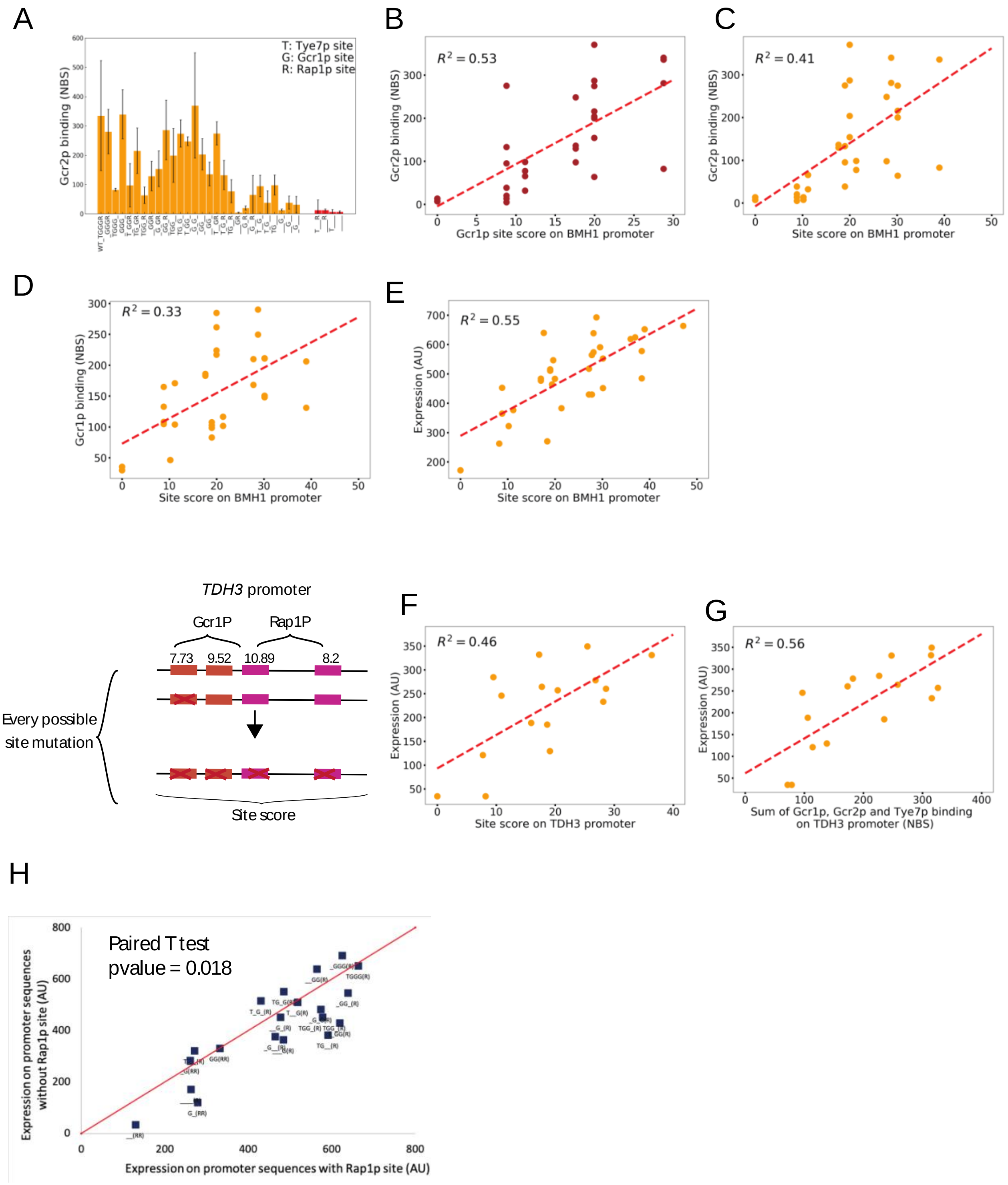

A
T: Tye7p site
G: Gcr1p site
R: Rap1p site
Gcr2p binding (NBS)
B
$R^2 = 0.53$
Gcr2p binding (NBS)
Gcr1p site score on BMH1 promoter
C
$R^2 = 0.41$
Gcr2p binding (NBS)
Site score on BMH1 promoter
D
$R^2 = 0.33$
Gcr1p binding (NBS)
Site score on BMH1 promoter
E
$R^2 = 0.55$
Expression (AU)
Site score on BMH1 promoter
TDH3 promoter
Gcr1P
Rap1P
7.73
9.52
10.89
8.2
Every possible site mutation
Site score
F
$R^2 = 0.46$
Expression (AU)
Site score on TDH3 promoter
G
$R^2 = 0.56$
Expression (AU)
Sum of Gcr1p, Gcr2p and Tye7p binding on TDH3 promoter (NBS)
H
Paired T test
pvalue = 0.018
Expression on promoter sequences without Rap1p site (AU)
Expression on promoter sequences with Rap1p site (AU)

Figure 7S. A) The same as in **Figure 5d** but with Gcr2p. **B)** The same as in **Figure 5e** but with Gcr2p. **C)** Gcr2p binding was compared to the total PWM scores from all remaining sites, which has weaker correlation than comparing to the total PWM scores from Gcr1/2p sites alone. **D)** The same as in **Figure 7CS** but with Gcr1p. **E)** Expression was regressed against the PWM scores from the remaining sites on *BMH1* promoter. **F)** We mutated *TDH3* promoter the same as we did for *BMH1* promoter, and expression was regressed against site score on TDH3 promoter. **G)** Gcr1p, Gcr2p and Tye7p binding were also measured for *TDH3* promoter, and we regressed the expression against the sum of all binding. **H)** Expression for sequences from *BMH1* and *TDH3* promoters is divided pairs that is either with or without any Rap1p motif. The red line is a 1:1 diagonal line for clear visualization purpose. The expression for sequences with Rap1p motif is generally higher than those without any Rap1p motif. Paired T test was performed, and the p-value is 0.018.

SUPPLEMENTAL TABLE 1

Description	Primers	Sections referred in Method and Materials	Variable region used in the study
Amplify synthetic promoter library.<br>Yellow colored region is 11 bp sub library index. Red colored region is TF barcode.	F<br>5'CTATAGGGCGAATTGGGTAC<br>CATGGTACATT3'<br>R<br>5'TATGTGATAATGCCAATGAGCTCCATTAC<br>NNNNGGCCGCTCGACCAGGAT3'	Synthetic promoter library amplification	CATGGTACATT<br>GAATGGAGTCT<br>TGGACTGGATT<br>ACGTTTGGGGT<br>ACCGAGGAAGT<br>CGTCGGCGATG<br>CATTAC<br>GTACGA<br>ACTGGT
Recover Ty5 Transposon inserted upstream of barcode and UMI in forward direction.<br>Orange colored is P5/P7, purple colored is read1/read2 seq primer.<br>Red colored region is barcode for multiplexing.	F<br>5'AATGATACGGCGACCACCGAGATCT<br>ACACTCTTTCCCTACACGACGCTCTTCCGATCT<br>GACATATAAGCTAGATCGTAATTCACTACGTCAACA3'<br>R<br>5'CAAGCAGAAGACGGCATACGAGAT<br>TTGTACATA<br>GTGACTGGAGTTCAGACGTGTGCTCTTCCGATCT<br>CCGGTGAACAGCTCCTCGCCCTT3'	Preparation of illumina libraries for calling card mapping	TTGTACATA<br>TCCACCTAG<br>ACATGTAGC<br>ACCCAGTCC<br>AGTTGGGCT<br>CGGGCAACG<br>TCCGATAAC<br>AGATGAGTG
Recover Ty5 Transposon inserted upstream of barcode and UMI in reverse direction.<br>Orange colored is P5/P7, purple colored is read1/read2 seq primer.<br>Red colored region is barcode for multiplexing.	F<br>5'AATGATACGGCGACCACCGAGATCT<br>ACACTCTTTCCCTACACGACGCTCTTCCGATCT<br>CAATTCCTGAAACAGTACGAAACATTACCC3'<br>R<br>5'CAAGCAGAAGACGGCATACGAGAT<br>TTGTACATA<br>GTGACTGGAGTTCAGACGTGTGCTCTTCCGATCT<br>CCGGTGAACAGCTCCTCGCCCTT3'	Preparation of illumina libraries for calling card mapping	TTGTACATA<br>TCCACCTAG<br>ACATGTAGC<br>ACCCAGTCC<br>AGTTGGGCT<br>CGGGCAACG<br>TCCGATAAC<br>AGATGAGTG

Recover Ty5 Transposon inserted downstream of barcode and UMI in forward direction.<br>Orange colored is P5/P7, purple colored is read1/read2 seq primer.<br>Red colored region is barcode for multiplexing.	F<br>5’AATGATACGGCGACCACCGAGATCT<br>ACACTCTTTCCCTACACGACGCTCTTCCGATCT<br>CTATAGGGCGAATTGGGTAC3’<br>5’CAAGCAGAAGACGGCATACGAGAT<br>TTGTACATA<br>GTGACTGGAGTTCAGACGTGTGCTCTTCCGATCT<br>CCCATATCATGCTTTTGGGTTATCACATTCAACA3’	Preparation of illumina libraries for calling card mapping	TTGTACATA<br>TCCACCTAG<br>ACATGTAGC<br>ACCCAGTCC<br>AGTTGGGCT<br>CGGGCAACG<br>TCCGATAAC<br>AGATGAGTG
Recover Ty5 Transposon inserted downstream of barcode and UMI in reverse direction.<br>Orange colored is P5/P7, purple colored is read1/read2 seq primer.<br>Red colored region is barcode for multiplexing.	F<br>5’AATGATACGGCGACCACCGAGATCT<br>ACACTCTTTCCCTACACGACGCTCTTCCGATCT<br>CTATAGGGCGAATTGGGTAC3’<br>5’CAAGCAGAAGACGGCATACGAGAT<br>TTGTACATA<br>GTGACTGGAGTTCAGACGTGTGCTCTTCCGATCT<br>GACATATAAGCTAGATCGTAATTCACTACGTCAACA3’	Preparation of illumina libraries for calling card mapping	TTGTACATA<br>TCCACCTAG<br>ACATGTAGC<br>ACCCAGTCC<br>AGTTGGGCT<br>CGGGCAACG<br>TCCGATAAC<br>AGATGAGTG
Obtain normalization factor	F<br>5’AATGATACGGCGACCACCGAGATCT<br>ACACTCTTTCCCTACACGACGCTCTTCCGATCT<br>CTATAGGGCGAATTGGGTAC3’<br>R<br>5’CAAGCAGAAGACGGCATACGAGAT<br>TTGTACATA<br>GTGACTGGAGTTCAGACGTGTGCTCTTCCGATCT<br>CCGGTGAACAGCTCCTCGCCCTT3’	Preparation of illumina libraries for calling card mapping	TTGTACATA<br>TCCACCTAG<br>ACATGTAGC<br>ACCCAGTCC<br>AGTTGGGCT<br>CGGGCAACG<br>TCCGATAAC<br>AGATGAGTG
Sort-seq library preparation	F<br>5’AATGATACGGCGACCACCGAGATCT<br>ACACTCTTTCCCTACACGACGCTCTTCCGATCT<br>CTATAGGGCGAATTGGGTAC3’<br>R<br>5’CAAGCAGAAGACGGCATACGAGAT<br>TTGTACATA<br>GTGACTGGAGTTCAGACGTGTGCTCTTCCGATCT<br>CCGGTGAACAGCTCCTCGCCCTT3’	Measuring reporter expression in CCRA libraries by Sort-seq	TTGTACATA<br>TCCACCTAG<br>ACATGTAGC<br>ACCCAGTCC<br>AGTTGGGCT<br>CGGGCAACG<br>TCCGATAAC<br>AGATGAGTG

SUPPLEMENTAL TABLE 2
Plasmids available in Addgene repository

Addgene ID	Plasmid	Note
**138616**	**Cbf1p_Sir4p**	**TF tagged with Sir4p for Calling Cards**
**138617**	**Tye7p_Sir4p**	**TF tagged with Sir4p for Calling Cards**
**138618**	**Gcr1p_Sir4p**	**TF tagged with Sir4p for Calling Cards**
**138619**	**Gcr2p_Sir4p**	**TF tagged with Sir4p for Calling Cards**
**138620**	**Gcn4p_Sir4p**	**TF tagged with Sir4p for Calling Cards**
**138621**	**Gal4p_Sir4p**	**TF tagged with Sir4p for Calling Cards**
**138622**	**Ty5_hisAI**	**Ty5 retrotransposon with inducible GAL promoter; an artificial intron is contained in the HIS selection marker**
**138623**	**YFP_TEF2pro_mCherry**	**Dual reporter backbone plasmid for synthetic promoter library cloning**

SUPPLEMENTAL NOTES

1. Plasmid map and sequences

a. Library backbone plasmid. Addgene ID 138623.

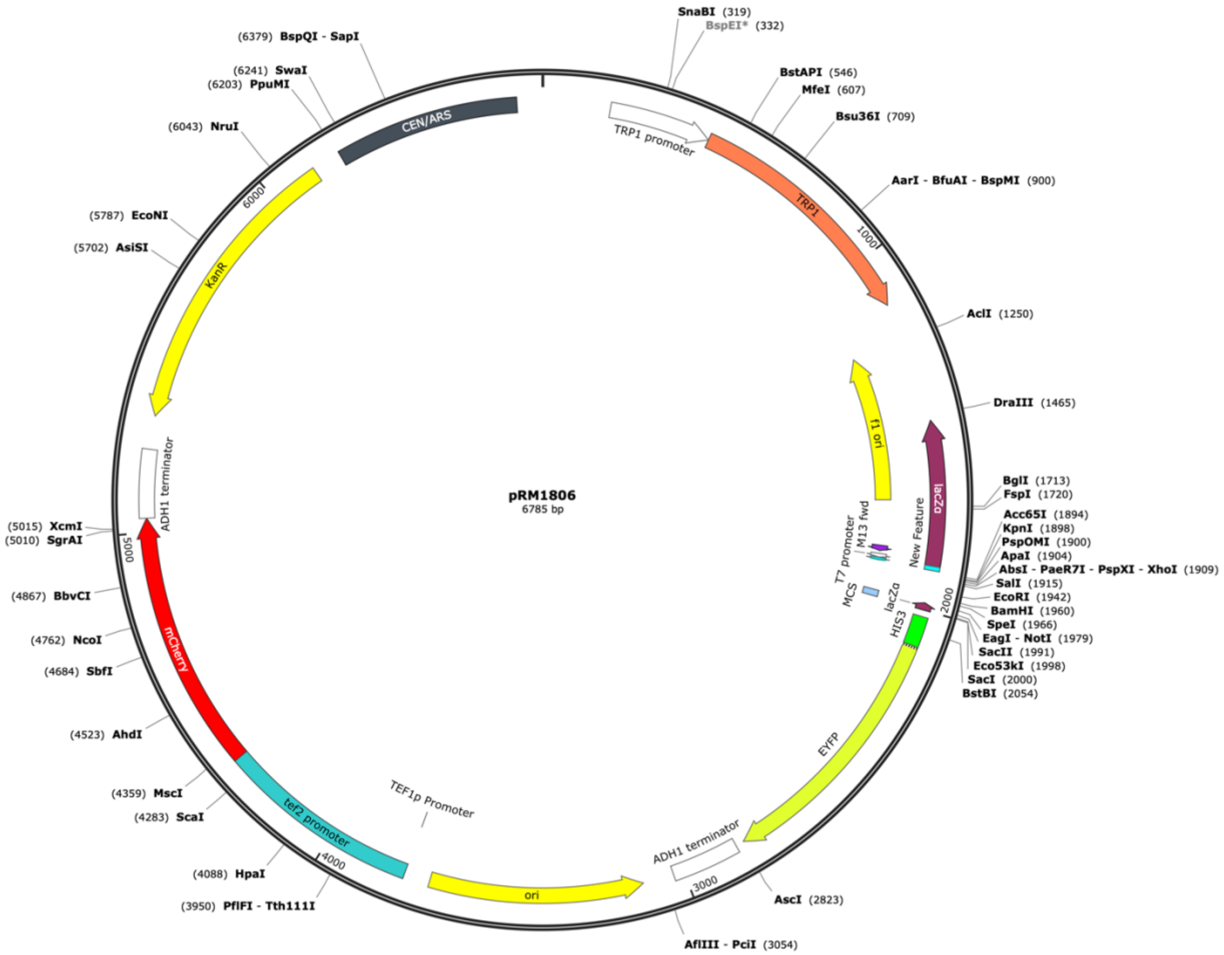

>pRM1806 (6785 bp)
TCGCGCGTTTCGGTGATGACGGTGAAAACCTCTGACACATGCAGCTCCCGGAGACGGTCACAGCTTGTCTGTAAGCGGATGCCGGGAGCAGACAAGCCCGTCAGGGCGCGTCAGCGGGTGTTGGCG
GGTGTCGGGGCTGGCTTAACTATGCGGCATCAGAGCAGATTGTACTGAGAGTGCACCATAAACGACATTACTATATATATAATATAGGAAGCATTTAATAGACAGCATCGTAATATATGTGTACTTTGC
AGTTATGACGCCAGATGGCAGTAGTGGAAGATATTCTTTATTGAAAAATAGCTTGTCACCTTACGTACAATCTTGATCCGGAGCTTTTCTTTTTTTGCCGATTAAGAATTAATTCGGTCGAAAAAAGAAA
AGGAGAGGGCCAAGAGGGAGGGCATTGGTGACTATTGAGCACGTGAGTATACGTGATTAAGCACACAAAGGCAGCTTGGAGTATGTCTGTTATTAATTTCACAGGTAGTTCTGGTCCATTGGTGAAA
GTTTGCGGCTTGCAGAGCACAGAGGCCGCAGAATGTGCTCTAGATTCCGATGCTGACTTGCTGGGTATTATATGTGTGCCCAATAGAAAGAGAACAATTGACCCGGTTATTGCAAGGAAAATTTCAAG
TCTTGTAAAAGCATATAAAAATAGTTCAGGCACTCCGAAATACTTGGTTGGCGTGTTTCGTAATCAACCTAAGGAGGATGTTTTGGCTCTGGTCAATGATTACGGCATTGATATCGTCCAACTGCATGG
AGATGAGTCGTGGCAAGAATACCAAGAGTTCCTCGGTTTGCCAGTTATTAAAAGACTCGTATTTCCAAAAGACTGCAACATACTACTCAGTGCAGCTTCACAGAAACCTCATTCGTTTATTCCCTTGTTT
GATTCAGAAGCAGGTGGGACAGGTGAACTTTTGGATTGGAACTCGATTTCTGACTGGGTTGGAAGGCAAGAGAGCCCCGAAAGCTTACATTTTATGTTAGCTGGTGGACTGACGCCAGAAAATGTTG
GTGATGCGCTTAGATTAAATGGCGTTATTGGTGTTGATGTAAGCGGAGGTGTGGAGACAAATGGTGTAAAAGACTCTAACAAAATAGCAAATTTCGTCAAAAATGCTAAGAAATAGGTTATTACTGAG
TAGTATTTATTTAAGTATTGTTTGTGCACTTGCCTATGCGGTGTGAAATACCGCACAGATGCGTAAGGAGAAAATACCGCATCAGGAAATTGTAAACGTTAATATTTTGTTAAAATTCGCGTTAAATTTT
TGTTAAATCAGCTCATTTTTTAACCAATAGGCCGAAATCGGCAAAATCCCTTATAAATCAAAAGAATAGACCGAGATAGGGTTGAGTGTTGTTCCAGTTTGGAACAAGAGTCCACTATTAAAGAACGTG
GACTCCAACGTCAAAGGGCGAAAAACCGTCTATCAGGGCGATGGCCCACTACGTGAACCATCACCCTAATCAAGTTTTTTGGGGTCGAGGTGCCGTAAAGCACTAAATCGGAACCCTAAAGGGAGCC
CCCGATTTAGAGCTTGACGGGGAAAGCCGGCGAACGTGGCGAGAAAGGAAGGGAAGAAAGCGAAAGGAGCGGGCGCTAGGGCGCTGGCAAGTGTAGCGGTCACGCTGCGCGTAACCACCACACC
CGCCGCGCTTAATGCGCCGCTACAGGGCGCGTCGCGCCATTCGCCATTCAGGCTGCGCAACTGTTGGGAAGGGCGATCGGTGCGGGCCTCTTCGCTATTACGCCAGCTGGCGAAAGGGGGATGTGCT
GCAAGGCGATTAAGTTGGGTAACGCCAGGGTTTTCCCAGTCACGACGTTGTAAAACGACGGCCAGTGAGCGCGCGTAATACGACTCACTATAGGGCGAATTGGGTACCGGGCCCCCCCTCGAGGTCG
ACGGTATCGATAAGCTTGATATCGAATTCCTGCAGCCCGGGGGATCCACTAGTTCTAGAGCGGCCGCCACCGCGGTGGAGCTCATTGGCATTATCACATAATGAATTATACATTATATAAAGTAATGTG
ATTTCTTCGAAGAATATACTAAAAAATGAGCAGGCAAGATAAACGAAGGCAAAGATGGTGAGCAAGGGCGAGGAGCTGTTCACCGGGGTGGTGCCCATCCTGGTCGAGCTGGACGGCGACGTAAAC
GGCCACAAGTTCAGCGTGTCCGGCGAGGGCGAGGGCGATGCCACCTACGGCAAGCTGACCCTGAAGTTCATCTGCACCACCGGCAAGCTGCCCGTGCCCTGGCCCACCCTCGTGACCACCTTCGGCTA
CGGCCTGCAGTGCTTCGCCCGCTACCCCGACCACATGAAGCAGCACGACTTCTTCAAGTCCGCCATGCCCGAAGGCTACGTCCAGGAGCGCACCATCTTCTTCAAGGACGACGGCAACTACAAGACCC

GCGCCGAGGTGAAGTTCGAGGGCGACACCCTGGTGAACCGCATCGAGCTGAAGGGCATCGACTTCAAGGAGGACGGCAACATCCTGGGGCACAAGCTGGAGTACAACTACAACAGCCACAACGTCT
ATATCATGGCCGACAAGCAGAAGAACGGCATCAAGGTGAACTTCAAGATCCGCCACAACATCGAGGACGGCAGCGTGCAGCTCGCCGACCACTACCAGCAGAACACCCCCATCGGCGACGGCCCCGT
GCTGCTGCCCGACAACCACTACCTGAGCTACCAGTCCGCCCTGAGCAAAGACCCCAACGAGAAGCGCGATCACATGGTCCTGCTGGAGTTCGTGACCGCCGCCGGGATCACTCTCGGCATGGACGAG
CTGTACAAGTAAGGCGCGCCACTTCTAAATAAGCGAATTTCTTATGATTTATGATTTTTATTATTAAATAAGTTATAAAAAAAATAAGTGTATACAAATTTTAAAGTGACTCTTAGGTTTTAAAACGAAAA
TTCTTATTCTTGAGTAACTCTTTCCTGTAGGTCAGGTTGCTTTCTCAGGTATAGTATGAGGTCGCTCTTATTGACCACACCTCTACCGGAATCAGGGGATAACGCAGGAAAGAACATGTGAGCAAAAGG
CCAGCAAAAGGCCAGGAACCGTAAAAAGGCCGCGTTGCTGGCGTTTTTCCATAGGCTCCGCCCCCCTGACGAGCATCACAAAAATCGACGCTCAAGTCAGAGGTGGCGAAACCCGACAGGACTATAA
AGATACCAGGCGTTTCCCCCTGGAAGCTCCCTCGTGCGCTCTCCTGTTCCGACCCTGCCGCTTACCGGATACCTGTCCGCCTTTCTCCCTTCGGGAAGCGTGGCGCTTTCTCATAGCTCACGCTGTAGGT
ATCTCAGTTCGGTGTAGGTCGTTCGCTCCAAGCTGGGCTGTGTGCACGAACCCCCCGTTCAGCCCGACCGCTGCGCCTTATCCGGTAACTATCGTCTTGAGTCCAACCCGGTAAGACACGACTTATCGC
CACTGGCAGCAGCCACTGGTAACAGGATTAGCAGAGCGAGGTATGTAGGCGGTGCTACAGAGTTCTTGAAGTGGTGGCCTAACTACGGCTACACTAGAAGGACAGTATTTGGTATCTGCGCTCTGCT
GAAGCCAGTTACCTTCGGAAAAAGAGTTGGTAGCTCTTGATCCGGCAAACAAACCACCGCTGGTAGCGGTGGTTTTTTTGTTTGCAAGCAGCAGATTACGCGCAGAAAAAAAGGATCTCAAGAAGATC
CTTTGATCTTTTCTACGGGGTCTGACGCTCAGTGGAACGAAAACTCACGTTAAGGGATTTTGGTCTATACTTACATATAGTAGATGTCAAGCGTAGGCGCTTCCCCTGCCGGCTGTGAGGGCGCCATAA
CCAAGGTATCTATAGACCGCCAATCAGCAAACTACCTCCGTACATTCATGTTGCACCCACACATTTATACACCCAGACCGCGACAAATTACCCATAAGGTTGTTTGTGACGGCGTCGTACAAGAGAACG
TGGGAACTTTTTAGGCTCACCAAAAAAGAAAGAAAAAAATACGAGTTGCTGACAGAAGCCTCAAGAAAAAAAAAAATTCTTCTTCGACTATGCTGGAGGCAGAGATGATCGAGCCGGTAGTTAACTATAT
ATAGCTAAATTGGTTCCATCACCTTCTTTTCTGGTGTCGCTCCTTCTAGTGCTATTTCTGGCTTTTCCTATTTTTTTTTTTCCATTTTTCTTTCTCTCTTTCTAATATATAAATTCTCTTGCATTTTCTATTTTTCT
CTCTATCTATTCTACTTGTTTATTCCCTTCAAGGTTTTTTTTTAAGGAGTACTTGTTTTTAGAATATACGGTCAACGAACTATAATTAACTAAACATGGTGAGCAAGGGCGAGGAGGATAACATGGCCATC
ATCAAGGAGTTCATGCGCTTCAAGGTGCACATGGAGGGCTCCGTGAACGGCCACGAGTTCGAGATCGAGGGCGAGGGCGAGGGCCGCCCCTACGAGGGCACCCAGACCGCCAAGCTGAAGGTGAC
CAAGGGTGGCCCCCTGCCCTTCGCCTGGGACATCCTGTCCCCTCAGTTCATGTACGGCTCCAAGGCCTACGTGAAGCACCCCGCCGACATCCCCGACTACTTGAAGCTGTCCTTCCCCGAGGGCTTCAA
GTGGGAGCGCGTGATGAACTTCGAGGACGGCGGCGTGGTGACCGTGACCCAGGACTCCTCCCTGCAGGACGGCGAGTTCATCTACAAGGTGAAGCTGCGCGGCACCAACTTCCCCTCCGACGGCCCC
GTAATGCAGAAGAAGACCATGGGCTGGGAGGCCTCCTCCGAGCGGATGTACCCCGAGGACGGCGCCCTGAAGGGCGAGATCAAGCAGAGGCTGAAGCTGAAGGACGGCGGCCACTACGACGCTGA
GGTCAAGACCACCTACAAGGCCAAGAAGCCCGTGCAGCTGCCCGGCGCCTACAACGTCAACATCAAGTTGGACATCACCTCCCACAACGAGGACTACACCATCGTGGAACAGTACGAACGCGCCGAG
GGCCGCCACTCCACCGGCGGCATGGACGAGCTGTACAAGTGAGCGAATTTCTTATGATTTATGATTTTTATTATTAAATAAGTTATAAAAAAAATAAGTGTATACAAATTTTAAAGTGACTCTTAGGTTT
TAAAACGAAAATTCTTATTCTTGAGTAACTCTTTCCTGTAGGTCAGGTTGCTTTCTCAGGTATAGTATGAGGTCGCTCTTATTGACCACACCTCTACCGGCACCTAGATCCCGGGGAAATGTGCGCGGAA
CCCCTATTTGTTTATTTTTCTAAATACATTCAAATATGTATCCGCTCATGAATTAATTCTTAGAAAAACTCATCGAGCATCAAATGAAACTGCAATTTATTCATATCAGGATTATCAATACCATATTTTTGAA
AAAGCCGTTTCTGTAATGAAGGAGAAAACTCACCGAGGCAGTTCCATAGGATGGCAAGATCCTGGTATCGGTCTGCGATTCCGACTCGTCCAACATCAATACAACCTATTAATTTCCCCTCGTCAAAAA
TAAGGTTATCAAGTGAGAAATCACCATGAGTGACGACTGAATCCGGTGAGAATGGCAAAAGTTTATGCATTTCTTTCCAGACTTGTTCAACAGGCCAGCCATTACGCTCGTCATCAAAATCACTCGCAT
CAACCAAACCGTTATTCATTCGTGATTGCGCCTGAGCGAGACGAAATACGCGATCGCTGTTAAAAGGACAATTACAAACAGGAATCGAATGCAACCGGCGCAGGAACACTGCCAGCGCATCAACAAT
ATTTTCACCTGAATCAGGATATTCTTCTAATACCTGGAATGCTGTTTTCCCGGGGATCGCAGTGGTGAGTAACCATGCATCATCAGGAGTACGGATAAAATGCTTGATGGTCGGAAGAGGCATAAATTC
CGTCAGCCAGTTTAGTCTGACCATCTCATCTGTAACATCATTGGCAACGCTACCTTTGCCATGTTTCAGAAACAACTCTGGCGCATCGGGCTTCCCATACAATCGATAGATTGTCGCACCTGATTGCCCG
ACATTATCGCGAGCCCATTTATACCCATATAAATCAGCATCCATGTTGGAATTTAATCGCGGCCTAGAGCAAGACGTTTCCCGTTGAATATGGCTCATAACACCCCTTGTATTACTGTTTATGTAAGCAG
ACAGTTTTATTGCACATTTCCCCGAAAAGTGCCACCTGGGTCCTTTTCATCACGTGCTATAAAAATAATTATAATTTAAATTTTTTAATATAAATATATAAATTAAAAATAGAAAGTAAAAAAAGAAATTA
AAGAAAAAATAGTTTTTGTTTTCCGAAGATGTAAAAGACTCTAGGGGGATCGCCAACAAATACTACCTTTTATCTTGCTCTTCCTGCTCTCAGGTATTAATGCCGAATTGTTTCATCTTGTCTGTGTAGAA
GACCACACACGAAAATCCTGTGATTTTACATTTTACTTATCGTTAATCGAATGTATATCTATTTAATCTGCTTTTCTTGTCTAATAAATATATATGTAAAGTACGCTTTTTGTTGAAATTTTTTAAACCTTTG
TTTATTTTTTTTTCTTCATTCCGTAACTCTTCTACCTTCTTTATTTACTTTCTAAAATCCAAATACAAAACATAAAAATAAATAAACACAGAGTAAATTCCCAAATTATTCCATCATTAAAAGATACGAGGCG
CGTGTAAGTTACAGGCAAGCGATCCGTCCTAAGAAACCATTATTATCATGACATTAACCTATAAAAATAGGCGTATCACGAGGCCCTTTCGTC

b. Ty5 transposon with an artificial intron. Addgene ID 138622.

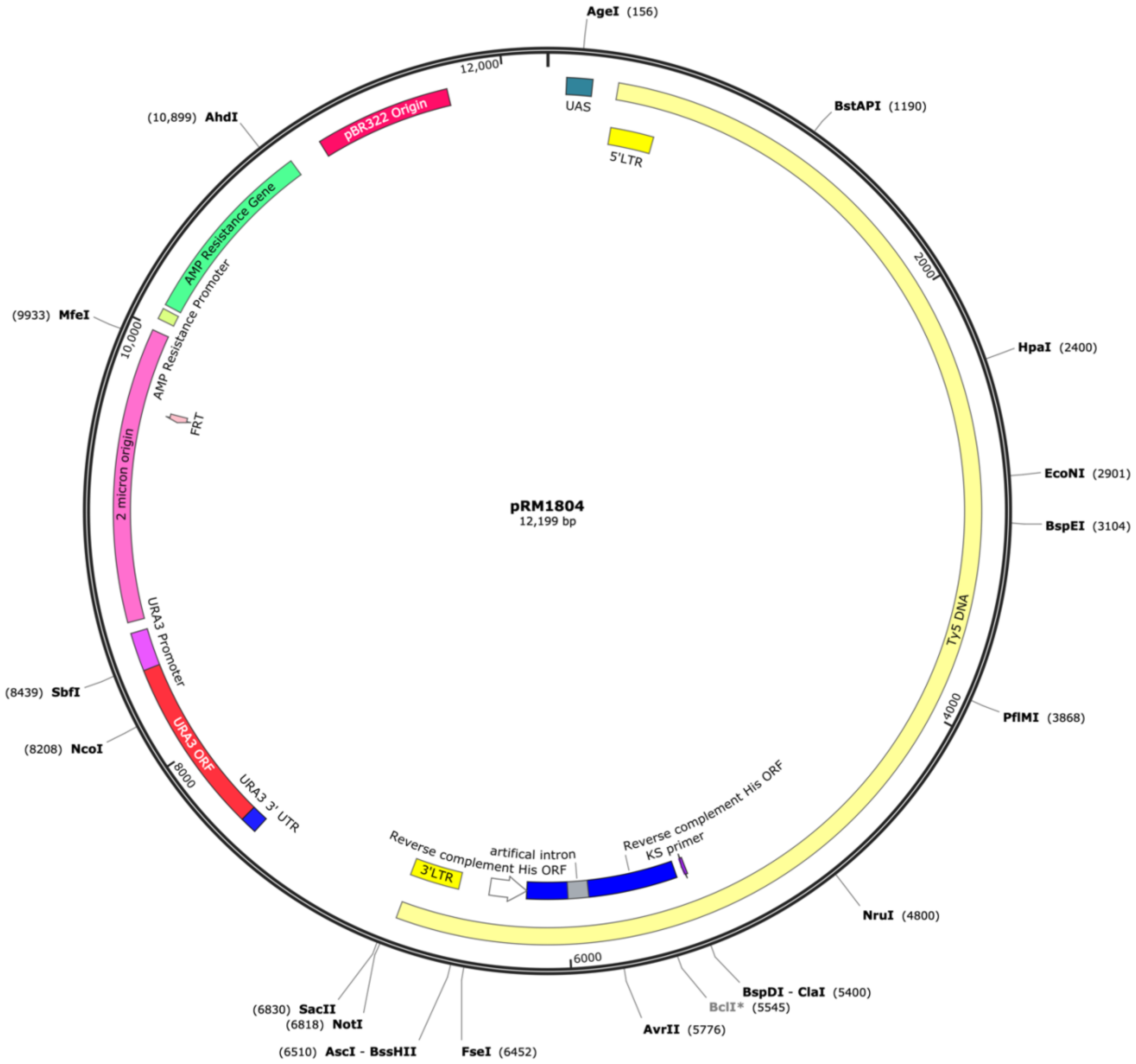

>pRM1804 (12,199 bp)
CTCGAGCCCCATTATCTTAGCCTAAAAAAACCTTCTCTTTGGAACTTTCAGTAATACGCTTAACTGCTCATTGCTATATTGAAGTACGGATTAGAAGCCGCCGAGCGGGTGACAGCCCTCCGAAG GAAG
ACTCTCCTCCGTGCGTCCTCGTCTTCACCGGTCGCGTTCCTGAAACGCAGATGTGCCTCGCGCCGCACTGCTCCGAACAATAAAGATTCTACAATACTAGCTTTTATGGTTATGAAGAGGAAAAA TTGGC
AGTAACCTGGCCCCACAAACCTTCAAATGAACGAATCAAATTAACAACCATAGGATGAGGGTAATGTTTCGTACTGTTTCAGGAATTGTTTCAGTAATGTTTTAGACAAGGAAAACATAGAGCAG CAAA
CCTCCGATCCGACAGTACTTAAGAAACCATAGTTTCTGTGTACAAGAGTAGTACCTATGTAATTCTTACATTTACATAACATATAGAAAGGTCCAATAAACTTACAACATTATGACATATAAGC TAGATC

GTAATTCACTACGTCAACAGGTTATGAGCCCTGAGAGCAATGCTTCAGAGACCATAATTAATCTATCTAATCCCAACAATTATAAACAGTGGCTGTACGGTATCGAGACCGCTGCTGAATATGCT AACG
AATATATGAACGAATTCGTTCATACCGGAGATATCCAATCAATGAAAAGGGATTGCAATCTCAGCGCGAATGATGAAAGCTTTGTCAAAACCGTATTTAACAGTTTCCTGGTAAAGCTCTA CAAGAAAA
CTATCGTGGGTGAAGCTGCATGTGAAATGAACTGGATATGTGATGATTCACTTGGAAGGGTCTCTGCTTATGATATTTTCTCGCACTTCGAAGAAAACTATTATGAAGTCACTAGTGGATCCAGG CTTA
CTCTTATAGAGGACTTACCAAATATATCCTCCAAGCCTGTAGATGAAATCGCTTCCTTTTTGAAAACCCTATTCACAATGCTTGAAGACAATAGCGAAGAACAGGACAAAAAGAAAAGA CGCGATACCA
ATATCGCGTTGCTATTAATGACCTTCTTACCCGAGTTAAAGGAATCATTCCACGAGAAATTCGGTGACTCTAAAGCTCTTCAGCTGTCACAAGTCATTAGATTCTGTAAATTAAAGGCGTCATCG AATTC
ATTATCTTCAGTCTCAGATGCATTGGTTGCACAAGACAGAAGAAGCTATCAAAAGAAAGGAAATAAGGGATGTATGATTTGTGGGGCTAATCATCGCTTAAGCAACTGTTCTCTGC TTAAAAGAAGAA
TACCAGAAGCCAGAATCTTTAAATTATATCCTAATGACAAGACGAATAGATCTTCATCTGCTAGTGTTGCGATTCCTGACTATGAAACGCAAGGCCAAACAGCAGGACAGATAACACCAAAGTCC TGGC
TCTGTATGTTATCTTCGACCGTCCCAGCTACCAAATCCTCAGATTGGATTTGTGACACAGGATGTACTTCACACATGTGCCACGACCGTTCTATGTTCTCATCATTTACTAGATC CTCTAAGAAAGACTTT
GTCAGAGGAGTCGGCGGTTCCATACCCATCATGGGCTCCGGGACTGTAAACATCGGCACTGTTCAATTAAATGACGTATCCTACGTCCCTGATTTACCAGTCAACCTAATATCCATTTGGAAACT ATGTG
CTAAATCCAACTCTTCTGTTACGTTCACAAAAGAGGGTGTCACTGTGAAATCACCTGATGACGTGATTTCTACGGCTGGGAAGTTAAACAATTATCTGTACATTTTCGAT GATCTTACGCCCGTAACTAC
CTTCTCTTCGCAAAATTACTTCTGCTCTAAAACATTGGATTCATCTAAAATGATAACTTCCGCAGCGTTTCATACCGTTGCAGATAAAATGTTGTCGCAACACATTTCTCCCACTGCTCTCCCGG TAAAATG
GCATGCTCGTATGGGCCATCCCGGAGCAGATATTTACAATTCCTTGGCTAGAACTCTGCGTTTTCCAAAATTTAAGACGGCTGAATACACTATTTGTCCTACCT GCTCACTAGCAAAAGGAATCATCAAA
AAGGGTAAAGTCTCGCTCAAAAAATATACCCAACCTCTTCAAATGGTACAGGCTGATCTCTGTGGTGGGTTTCGCTACCAAGAGTTTCAGTCAAATAAATATTTTCTTACTATCCGTGATGCCTA TAGTC
GCTACTACTCTGTAATACATTTAAAATCCAAAGCAGACGCTCCGATAAAATTCATGGAATGGATCAACGAAACCGAACAATACTTTAGCTCCCGGGGTGG ATTCAAAGTCGGATCTGTTCGTACAGACA
ATGGTACAGAATTCGTAAATAAAAATCTTCATGCGTTTTTAAATCTAAAGGAATAGAGCATCAGTTAACTATTCCATATCATAGTTATCAAAATGGTGCTGTTGAACGTGCACATCGTACCATC GAAGA
ACGCACTCGTTGTCTCCTTATCGGGGGGCGTGTTCCTCCGTCCTTGTGGTCTGAAGCTGTTTCTTGCGCAGTCTATTTAATCAATAGGTCCCCTGTA GTGTCCAAAAATAACAGTATCCCATACTGCCGGT
GGTTCAACATCCCCGCAAAAGATTTCGGTATCGCACATCTTCGAATTTTTGGATGTACAGCATACGCAACCTTACAACCTAGTCTTCGAGACGGCAAACTTGCCCCAACTGTCATATCTGGTGTT ATGGT
TGGCTATGACTCTAACCATCGAGGATACAGGATTTATCATCCCGAAACTGGCCGCATCTTTGTGAGCAGTCAAGTTCGATTTGACGAACACA TGTTTCCTCTTGCTGATACAGAGGCAGTTCACGTCTCT
CACGACTTTGCCACTTCCGCTATTGGGGGGGTGTCCAAATATCCTGAAACAGGGTCAACCGTCTCTGCTCCAAAGAACGACGGATCTGACTTGGCAAATTTGCCAATAACTGTTCCCAAAAATGT AAAT
CAACCAGCACATAAACCTAATACCAGTAACATCTCTTCCTCTGATGATGATGAGGATATTTCAATGGAAATCGAAATGGAAAAACCTAT CCCTGAGTGTAACCAAGACAACTTACCAAACTCCGGATGT
CCACCAACAAGGATACAACATTCTAACTTTGAATCCTTACCAACCGTGTCTACCGAAGACGAAACTAATTCTTCTATGGAGAAAACTCCTGAAAGAGTTCCAGCGGCACTAACTTATCGAGAAAT TCCAA
AATCATCCGATTCAGAATATATTCCGACATGCCGAAATAGAACTAGACGTGTTAAAAGAACTAATAAGAAACCAACGCGATCCCGC GAAATAGAAATATATGATATATCACGTCCAAACGTAATATCGA
GTGACAACTTACCTGAAGTTAGAAGTGCCAAGCAAAGAAAGACGGTGTCCAATACAAATGATACTGTAGCAAGGACAAATAGACTTCCAACCGTGCTACGAACTCTAGACTCAAACAACATTGAC ACG
CTGCATGTTGCCAGTACTGGTGAAGAAGTGTCCATCGAAAGACTTTCAAGCATGGCTCTTCAGGAAGCGAAGAACAATTCCGCCA GAACTAATCAAGCTAATTCTCTTACTGATTGGTTTCCAGTAGGC
GCAATGCCGATACCTGACCAGAGGTATCTATCCGTTCACGATGGAACATATATCAGCGACTCACAAGATGTGGGTGATACTGACCTCACTCCTGCTGTAACCAGGCTAGTTACTGAAGAGAATTC AATC
GAATCTCCTCCATCGTTGGATTCATCGCCTCCAAATACCTCATTTAACGCGGCTCTAACTGCTATTATCCATAGCACAAAAAA AGGAAACCCGAAAACCTATGCCCAAGCAATGGGAAGGCCTGACTTTC
AAGAATGGCACAACGCATGCCTCAAGGAACTTTCCGCGTTCAAAGATCACAATACGTACAAATTGGTGTCTCTTCCAAAGCAAAGAAGAGCTCTTGGATCGCGCTGGGTATTCACAATAAAAGAC TCC
GGGACGTACAAAGCTCGCCTTGTCGCCCAAGGACATACTCAAAAGGCTGGTATTGACTATCAAGAAACTTTTGCACCAGTCATTCGATATGACTCTGTTAGATTATTTCTGGCCCTTGCTAGCTGCCTCA
AACTAATAGTATATCAGATGGACGTTGACACCGCGTTTCTAAACTCAAAAATGAATGAGCCGGTATACGTAAAACAACCACCCGGATTTATTAATGAAAGTAATCCCGACTATGTATGGGAACTA TACG
GCGGTATGTATGGACTCAAGCAAGCCCCATTACTATGGAACGAACATATCAACAATACTCTTCAAAAGATTGGTTTTC GTCGACATGAAGGCGAACATGGCTTATACTTTCGTTCCACATCTGATGGTCC
CATCTACATTGCCCTATACGTAGACGACTTACTTGTTGCTGCTCCCTCTCCGAAAATATATGACAGGGTTAAGCAGAAACTAACGAAGTTATACTCAATGAAGGATCTAGGTAAAGTTGACAAAT TCCTC
GGTCTTAACATTAATCAATTTTCAAATGGAGACATCACTCTCTCACTTCAAGACTATATTGCTAAAGCTGCATCTGAAAGCGAAATAAACATATGTAAGCCTACACAGACTCCGCTCTGTGACTCAAAGC
CTCTTTTCGAAACAACTTCCCCGCACCTAAAGGACATCACTCCTTATCAGAGCATAGTTGGACAGCTTCTCTTTTGTGCAAATACTGGTCGTCCTGACATATCTTATCCGGTCTCACTACTCTCC AGGTTTC
TTCGCGAACCTCGCGCAATCCATTTGGAGTCTGCTCGACGAGTTCTACGGTACCTATATACCACCAGAAGTATGTGTCTCAAGTATCGTTCTGGATCTCTGTTGGCACTAACTGTATATTGTGATGCATCT
CATGGAGCAATTCACGATCTCCCACACTCTACTGGGGGGTACGTGACTCTACTTGCTGGTGCTCCAGTTACGTGGTCATCAAAGAAACTCAAGGGTGTGATTCCTGTATCATCTACTGAGGCAGA ATAC
ATTACTGCAAGTGAAACTGTCATGGAGATAGAATGGATTCAAAACTTGTTTGAACACTTAGGCCAGCCACTTATCTCATCAACATTATACGTAGATAATGAACCTGCTATAAAACTATCTAAACATCCTG
TATTTCACACGAGAACAAAACACATTGCCTTGAGATATCACAAGCTAAGAAGTGCAGTGGCAGCAGGCATAATTACCATAGAGCATGTTATTACAAAGAGACAAGTTGCTGACATATTTACAAAA ATCC
TTCCAGCAGAATCATTCAAAGCACATAGGGCTGTCATGGTGAGGGAACCAGAAACTGCAAAATAACAGATCGAGGTCGACGGTATCGATAAGCTTGGGCTGCAGCTTTAAATAATCGGTGTCACTACA
TAAGAACACCTTTGGTGGAGGGAACATCGTTGGTACCATTGGGCGAGGTGGCTTCTCTTATGGCAACCGCAAGAGCCTTGAACGCACTCTCACTACGGTGATGATCATTCTTGCCTCGCAGACAA TCAA
CGTGGAGGGTAATTCTGCTAGCCTCTGCAAAGCTTTCAAGAAAATGCGGGATCATCTCGCAAGAGAGATCTCCTACTTTCTCCCTTTGCAAACCAAGTTCGACAACTGCGTACGGCCTGTTCGAAAGAT
CTACCACCGCTCTGGAAAGTGCCTCATCCAAAGGCGCAAATCCTGATCCAAACCTTTTTACTCCACGCACGGCCCCTAGGGCCTCTTTAAAAGCTTGACCGAGAGCAATCCCGCAGTCTTCAGTG GTGT
GATGGTCGTCTATGTGTAAGTCACCAATGCACTCAACGATTAGCGACCAGCCGGAATGCTTGGGTATGTTAATATGGACTAAAGGAGGCTTTTCTGCAGGTCGACTCTAGAGGATCCCCGGGTACCGA
GCTCGAATTTTTACTAACAAATGGTATTATTTATAACAGCCAGAGCATGTATCATATGGTCCAGAAACCCTATACCTGTGTGGACGTTAATCACTTGCGATTGTGTGGCCTGTTCTGCTACTGCT TCTGCC
TCTTTTTCTGGGAAGATCGAGTGCTCTATCGCTAGGGGACCACCCTTTAAAGAGATCGCAATCTGAATCTTGGTTTCATTTGTAATACGCTTTACTAGGGCTTTCTGCTCTGTCATCTTTGCCTTCGTTTAT
CTTGCCTGCTCATTTTTTAGTATATTCTTCGAAGAAATCACATTACTTTATATAATGTATAATTCATTATGTGATAATGCCAATCGCTAAGAAAAAAAAAGAGTCATCCGCTAGGTGGAAAAAAA AAAAT
GAAAATCATTACCGAGGCATAAAAAAATATAGAGTGTACTAGAGGCTCCAAGAGTGTACTAGAGGATCCCCGGGCGAGCTCGAATTCCGAGCTTAGGCCGGCCTCGAGTCGGAGGCCAAGGCGGCC
GTACGTCCAACCCTATACTGAAACTGACGCAGGCGCGCCGGGATCTCATGCGTATTCAGTTATGGGGGGATGTTGAATGTGATAACCCAAAAGCATGATATGGGTAATGTTTCAGTACTGTTTCA GAA
TTGTTTCAGTAATGTTTTAGACAAGGAAAACATAGAGCAGCAAACCTCTGATCCGACAGTACTTAAGAAACCATAGTTTCTGTGTAGAAGAGTAGTACCTATGTAATTCTTACATTTACATAACATATAG
AAAGGTCCAATAAACTTACAACATTATGACATATAAGCTAGATCGTAATTCACTACGTCAACAGGATCCACTAGTTCTAGAGCGGCCGCCACCGCGGTGGAGCTCCAATTCGCCCTATAGTGAGT CGTA
TTACAATTCACTGGCCGTCGTTTTACAACGTCGTGACTGGGAAAACCCTGGCGTTACCCAACTTAATCGCCTTGCAGCACATCCCCCTTTCGCCAGCTGGCGTAATAGCGAAGAGGCCCGCACCGATCG
CCCTTCCCAACAGTTGCGCAGCCTGAATGGCGAATGGACGCGCCCTGTAGCGGCGCATTAAGCGCGGCGGGTGTGGTGGTTACGCGCAGCGTGACCGCTACACTTGCCAGCGCCCTAGCGCCCGC TC
CTTTCGCTTTCTTCCCTTCCTTTCTCGCCACGTTCGCCGGCTTTCCCCGTCAAGCTCTAAATCGGGGGCTCCCTTTAGGGTTCCGATTTAGTGCTTTACGGCACCTCGACCCCAAAAAACTTGATTAGGGTG
ATGGTTCACGTAGTGGGCCATCGCCCTGATAGACGGTTTTTCGCCCTTTGACGTTGGAGTCCACGTTCTTTAATAGTGGACTCTTGTTCCAAACTGGAACAACACTCAACCCTATCTCGGTCTAT TCTTTT
GATTTATAAGGGATTTTGCCGATTTCGGCCTATTGGTTAAAAAATGAGCTGATTTAACAAAAATTTAACGCGAATTTTAACAAAATATTAACGTTTACAATTTCCTGATGCGGTATTTTCTCCTTACGCAT
CTGTGCGGTATTTCACACCGCAGGGTAATAACTGATATAATTAAATTGAAGCTCTAATTTGTGAGTTTAGTATACATGCATTTACTTATAATACAGTTTTTTAGTTTTGCTGGCCGCATCTTCTC AAATATG
CTTCCCAGCCTGCTTTTCTGTAACGTTCACCCTCTACCTTAGCATCCCTTCCCTTTGCAAATAGTCCTCTTCCAACAATAATAATGTCAGATCCTGTAGAGACCACATCATCCACGGTTCTATACTGTTGACC
CAATGCGTCTCCCTTGTCATCTAAACCCACACCGGGTGTCATAATCAACCAATCGTAACCTTCATCTCTTCCACCCATGTCTCTTTGAGCAATAAAGCCGATAACAAAATCTTTGTCGCTCTTCG CAATGTC
AACAGTACCCTTAGTATATTCTCCAGTAGATAGGGAGCCCTTGCATGACAATTCTGCTAACATCAAAAGGCCTCTAGGTTCCTTTGTTACTTCTTCTGCCGCCTGCTTCAAACCGCTAACAATACCTGGGC
CCACCACACCGTGTGCATTCGTAATGTCTGCCCATTCTGCTATTCTGTATACACCCGCAGAGTACTGCAATTTGACTGTATTACCAATGTCAGCAAATTTTCTGTCTTCGAAGAGTAAAAAATTG TACTTG
GCGGATAATGCCTTTAGCGGCTTAACTGTGCCCTCCATGGAAAAATCAGTCAAGATATCCACATGTGTTTTTAGTAAACAAATTTTGGGACCTAATGCTTCAACTAACTCCAGTAATTCCTTGGTGGTAC
GAACATCCAATGAAGCACACAAGTTTGTTTGCTTTTCGTGCATGATATTAAATAGCTTGGCAGCAACAGGACTAGGATGAGTAGCAGCACGTTCCTTATATGTAGCTTTCGACATGATTTATCTT CGTTT
CCTGCAGGTTTTTGTTCTGTGCAGTTGGGTTAAGAATACTGGGCAATTTCATGTTTCTTCAACACTACATATGCGTATATATACCAATCTAAGTCTGTGCTCCTTCCTTCGTTCTTCCTTCTGTTCGGAGAT
TACCGAATCAAAAAAATTTCAAAGAAACCGAAATCAAAAAAAAGAATAAAAAAAAAAATGATGAATTGAATTGAAAAGCGGAAGCATCTGTGCTTCATTTTGTAGAACAAAAATGCAACGCGAGAG CG
CTAATTTTTCAAACAAAGAATCTGAGCTGCATTTTTACAGAACAGAAATGCAACGCGAAAGCGCTATTTTACCAACGAAGAATCTGTGCTTCATTTTTGTAAAACAAAAATGCAACGCGAGAGCGCTAA
TTTTTCAAACAAAGAATCTGAGCTGCATTTTTACAGAACAGAAATGCAACGCGAGAGCGCTATTTTACCAACAAAGAATCTATACTTCTTTTTTGTTCTACAAAAATGCATCCCGAGAGCGCTAT TTTTCT
AACAAAGCATCTTAGATTACTTTTTTTCTCCTTTGTGCGCTCTATAATGCAGTCTCTTGATAACTTTTTGCACTGTAGGTCCGTTAAGGTTAGAAGAAGGCTACTTTGGTGTCTATTTTCTCTTCCATAAAA
AAAGCCTGACTCCACTTCCCGCGTTTACTGATTACTAGCGAAGCTGCGGGTGCATTTTTTCAAGATAAAGGCATCCCCGATTATATTCTATACCGATGTGGATTGCGCATACTTTGTGAACAGAA AGTGA
TAGCGTTGATGATTCTTCATTGGTCAGAAAATTATGAACGGTTTCTTCTATTTTGTCTCTATATACTACGTATAGGAAATGTTTACATTTTCGTATTGTTTTCGATTCACTCTATGAATAGTTCTTA CTACAA
TTTTTTTGTCTAAAGAGTAATACTAGAGATAAACATAAAAAATGTAGAGGTCGAGTTTAGATGCAAGTTCAAGGAGCGAAAGGTGGATGGGTAGGTTATATAGGGATATAGCACAGAGATATATA GC
AAAGAGATACTTTTGAGCAATGTTTGTGGAAGCGGTATTCGCAATATTTTAGTAGCTCGTTACAGTCCGGTGCGTTTTTGGTTTTTTGAAAGTGCGTCTTCAGAGCGCTTTTGGTTTTCAAAAGC GCTCT
GAAGTTCCTATACTTTCTAGAGAATAGGAACTTCGGAATAGGAACTTCAAAGCGTTTCCGAAAACGAGCGCTTCCGAAAATGCAACGCGAGCTGCGCACATACAGCTCACTGTTCACGTCGCAC CTATA
TCTGCGTGTTGCCTGTATATATATATACATGAGAAGAACGGCATAGTGCGTGTTTATGCTTAAATGCGTACTTATATGCGTCTATTTATGTAGGATGAAAGGTAGTCTAGTACCTCCTGTGATAT TATCC
CATTCCATGCGGGGTATCGTATGCTTCCTTCAGCACTACCCTTTAGCTGTTCTATATGCTGCCACTCCTCAATTGGATTAGTCTCATCCTTCAATGCTATCATTTCCTTTGATATTGGATC AGGCACTTTTCG
GGGAAATGTGCGCGGAACCCCTATTTGTTTATTTTTCTAAATACATTCAAATATGTATCCGCTCATGAGACAATAACCCTGATAAATGCTTCAATAATATTGAAAAAGGAAGAGTATGAGTATTC AACAT
TTCCGTGTCGCCCTTATTCCCTTTTTTGCGGCATTTTGCCTTCCTGTTTTTGCTCACCCAGAAACGCTGGTGAAAGTAAAAGATGCTGAAGATCAGTTGGGTGCACGAGTGGGT TACATCGAACTGGATC
TCAACAGCGGTAAGATCCTTGAGAGTTTTCGCCCCGAAGAACGTTTTCCAATGATGAGCACTTTTAAAGTTCTGCTATGTGGCGCGGTATTATCCCGTATTGACGCCGGGCAAGAGCAACTCGGT CGCC
GCATACACTATTCTCAGAATGACTTGGTTGAGTACTCACCAGTCACAGAAAAGCATCTTACGGATGGCATGACAGTAAGAGAATTATGCAGTGCTGCCATAACCATGAGTG ATAACACTGCGGCCAAC
TTACTTCTGACAACGATCGGAGGACCGAAGGAGCTAACCGCTTTTTTTCACAACATGGGGGATCATGTAACTCGCCTTGATCGTTGGGAACCGGAGCTGAATGAAGCCATACCAAACGACGAGCG TGA
CACCACGATGCCTGTAGCAATGGCAACAACGTTGCGCAAACTATTAACTGGCGAACTACTTACTCTAGCTTCCCGGCAACAATTAATAGACTGGATGGAGGCGGATAAAGT TGCAGGACCACTTCTGC
GCTCGGCCCTTCCGGCTGGCTGGTTTATTGCTGATAAATCTGGAGCCGGTGAGCGTGGGTCTCGCGGTATCATTGCAGCACTGGGGCCAGATGGTAAGCCCTCCCGTATCGTAGTTATCTACACG ACG
GGCAGTCAGGCAACTATGGATGAACGAAATAGACAGATCGCTGAGATAGGTGCCTCACTGATTAAGCATTGGTAACTGTCAGACCAAGTTTACTCATATATACTTTAGATT GATTTAAAACTTCATTTTT

AATTTAAAAGGATCTAGGTGAAGATCCTTTTTGATAATCTCATGACCAAAATCCCTTAACGTGAGTTTTCGTTCCACTGAGCGTCAGACCCCGTAGAAAAGATCAAAGGATCTTCTTGAGATCCTTTTTTT
CTGCGCGTAATCTGCTGCTTGCAAACAAAAAAACCACCGCTACCAGCGGTGGTTTGTTTGCCGGATCAAGAGCTACCAACTCTTTTTCCGAAGGTAACTGGCTTCAGCAGAGCGCAGATACCAAATACT
GTCCTTCTAGTGTAGCCGTAGTTAGGCCACCACTTCAAGAACTCTGTAGCACCGCCTACATACCTCGCTCTGCTAATCCTGTTACCAGTGGCTGCTGCCAGTGGCGATAAGTCGTGTCTTACCGGGTTGG
ACTCAAGACGATAGTTACCGGATAAGGCGCAGCGGTCGGGCTGAACGGGGGGTTCGTGCACACAGCCCAGCTTGGAGCGAACGACCTACACCGAACTGAGATACCTACAGCGTGAGCATTGAGAAA
GCGCCACGCTTCCCGAAGGGAGAAAGGCGGACAGGTATCCGGTAAGCGGCAGGGTCGGAACAGGAGAGCGCACGAGGGAGCTTCCAGGGGGGAACGCCTGGTATCTTTATAGTCCTGTCGGGTTT
CGCCACCTCTGACTTGAGCGTCGATTTTTGTGATGCTCGTCAGGGGGGCCGAGCCTATGGAAAAACGCCAGCAACGCGGCCTTTTTACGGTTCCTGGCCTTTTGCTGGCCTTTTGCTCACATGTTCTTTC
CTGCGTTATCCCCTGATTCTGTGGATAACCGTATTACCGCCTTTGAGTGAGCTGATACCGCTCGCCGCAGCCGAACGACCGAGCGCAGCGAGTCAGTGAGCGAGGAAGCGGAAGAGCGCCCAATACG
CAAACCGCCTCTCCCCGCGCGTTGGCCGATTCATTAATGCAGCTGGCACGACAGGTTTCCCGACTGGAAAGCGGGCAGTGAGCGCAACGCAATTAATGTGAGTTACCTCACTCATTAGGCACCCCAGG
CTTTACACTTTATGCTTCCGGCTCCTATGTTGTGTGGAATTGTGAGCGGATAACAATTTCACACAGGAAACAGCTATGACCATGATTACGCCAAGCTCGGAATTAACCCTCACTAAAGGGAACAAAAGC
TGGGTACCGGGCCCCCC

2. Library design and cloning

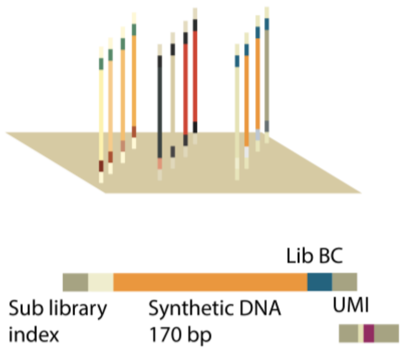

The designed synthetic promoter library was synthesized by Aglient. The maximum length of oligo is 230bp, and the library have up to 244,000 unique sequences.

One example from library design:
CTATAGGGCGAATTGGGTACCATGGTACATTTGACCTATGACGCCCGATGCTCGGAGAAGGTGGTCAG
GCACACGTGATTCACCTAAACTCGGCGCCGAAAAAAATTTGATCAGCCGTACAGTGTGATTGTTATCACG
TGATAGTAACGTAAGTCACGTGCTTTCTAACCATCACCCCGCACGAACGTTCGTTCCCGTTTCGTTTCATC
ACGGATCCTGGTCGAGCGGCC

library barcode

Forward primer for library amplification

11 bp sub library index

5’- CTATAGGGCGAATTGGGTACCATGGTACATT-3’

Reverse primer for library amplification

3’-ATCCTGGTCGAGCGGCCNNNNGTAATGGAGCTCATTGGCATTATCACATA-5’

UMI TF barcode

PCR product and ready for Gibson cloning with the linearized plasmid backbone
5’-
CTATAGGGCGAATTGGGTACCATGGTACATTTGACCTATGACGCCCGATGCTCGGAGAAGGTGGTCAG

GCACACGTGATTCACCTAAACTCGGCGCCGAAAAAAATTTGATCAGCCGTACAGTGTGATTGTTATCACG
TGATAGTAACGTAAGTCACGTGCTTTCTAACCATCACCCCGCACGAACGTTCGTTCCCGTTTCGTTTCATC
ACGGATCCTGGTCGAGCGGCCNNNNGTAATGGAGCTCATTGGCATTATCACATA-3’

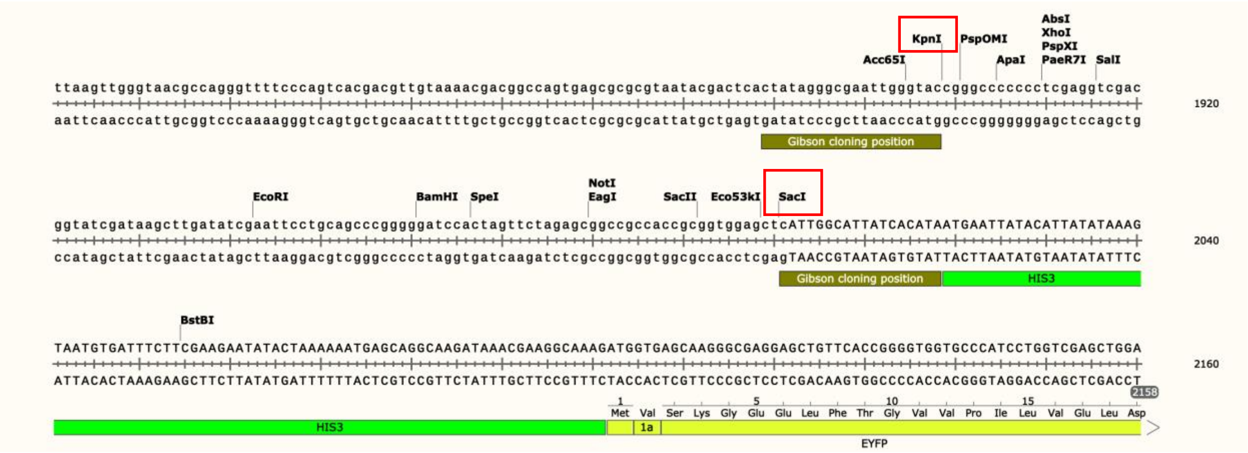

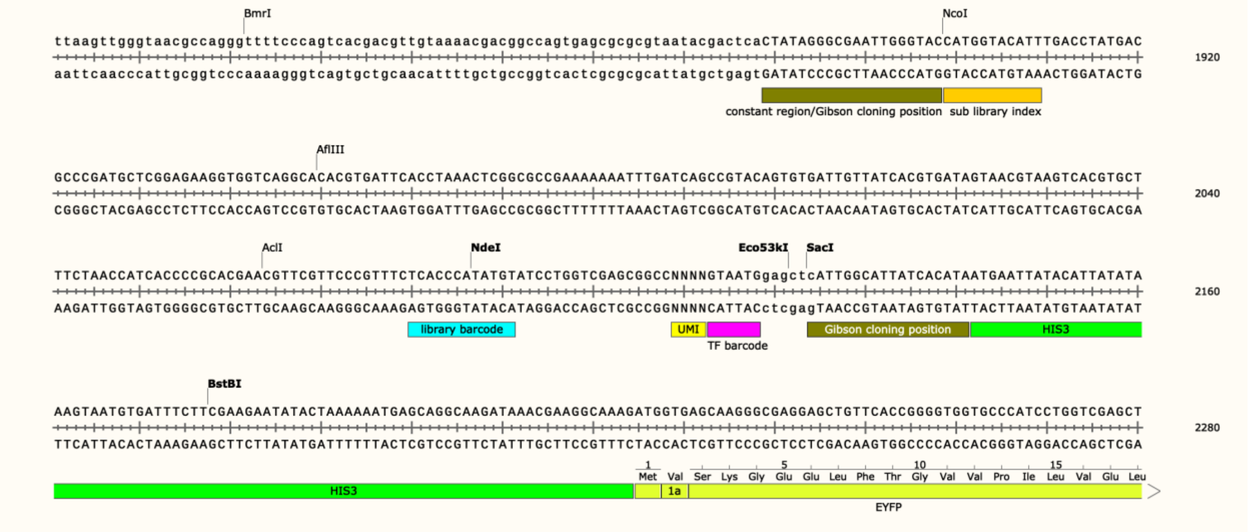

3. **Transposition recovery from sequencing reads demonstration**

Sequencing reads example for each PCR product:

12mer Library barcode

4mer UMI

6mer TF barcode

Forward primer used for sequencing

Reverse primer used for sequencing

Red colored DNA sequences are used for mapping the insertion position

1) Ty5 Transposon inserted upstream of barcode and UMI in forward direction

1 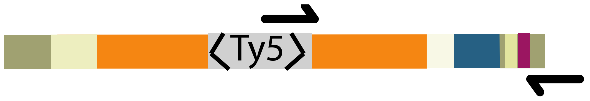

Ty5 inserted in forward direction

Ty5 3'LTR

R1
GACATATAAGCTAGATCGTAATTCACTACGTCAACACAATAAACATATAATAAATTCCTTATCTGGGTAG
GTGTCTGGGTGAACAGGTGTGTCCTGGCATCCACTAAAGTAAGCGAAACTCAAATTGAGGGCTGGCATC
CAGAAATTTTTTTTTCTTCTACAACTCCCGGATCCTGGTCGAGCGGCCTAGGTCGTACGAGCTCATTGGC
ATTATCACATAATGAATTATACATTATATAAAGTAATGTGAT

R2(Reverse complement)
GGTGTGTCCTGGCATCCACTAAAGTAAGCGAAACTCAAATTGAGGGCTGGCATCCAGAAATTTTTTTTTC
TTCTACAACTCCCGGATCCTGGTCGAGCGGCCTAGGTCGTACGAGCTCATTGGCATTATCACATAATGAA
TTATACATTATATAAAGTAATGTGATTTCTTCGAAGAATATACTAAAAAATGAGCAGGCAAGATAAACGA
AGGCAAAGATGGTGAGCAAGGGCGAGGAGCTGTTCACCGG

Mapped library reference sequence
ccgctacagggcgcgtcgcgccattcgccattcaggctgcgcaactgttgggaagggcgatcggtgcgggcctcttcgctattacgccag
ctggcgaaagggggatgtgctgcaaggcgattaagttgggtaacgccagggttttcccagtcacgacgttgtaaaacgacggccagtga
gcgcgcgtaatacgactcaCTATAGGGCGAATTGGGTACTGGACTGGATTCGATAATTTTCATTTTTTTTTTTCT
GTAACCCGTACATGCCCTTTTTACAATAAACATATAATAAATCCCTTATCTGGGTAGGTGTCTGGGTGAA
CAGGTGTGTCCTGGCATCCACTAAAGTAAGCGAAACTCAAATTGAGGGCTGGCATCCAGAAATTTTTTTT
TCTTCTACAACTCCCGGATCCTGGTCGAGCGGCCNNNNTCGTACgagctcATTGGCATTATCACATAATGA
ATTATACATTATATAAAGTAATGTGATTTCTTCGAAGAATATACTAAAAAATGAGCAGGCAAGATAAACG
AAGGCAAAGATGGTGAGCAAGGGCGAGGAGCTGTTCACCGGGGTGGTGCCCATCCTGGTCGAGCTGG
ACGGCGACGTAAACGGCCACAAGTTCAGCGTGTCCGGCGAGGGCGAGGGCGATGCCACCTACGGCAA
GCT

2) Ty5 Transposon inserted upstream of barcode and UMI in reverse direction

2

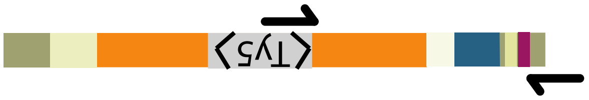

Ty5 inserted in reverse direction

Ty5 5’LTR (reverse complement)

R1
CCCATATCATGCTTTTGGGTTATCACATTCAACAGACTGGATTGAAACGGGAACGAACGAGCCACGTGA
CGGGCTTCCTCTTAGTACGATACTTACGTTACTATTCAGTGATAACAATCACACTGTACGGCTGATCAGA
GGAAGCTCGGTGGCAAATAGCTTCTCTTACTGGCCGGGGCGCGGGGGGACCGAGCATCGGGCGTCATA
GGTCAAGGGTTTTCGTCATCCTGGTCGAGCGGCCCTGCTCGTA

R2(Reverse complement)
GGAAGCTCGGTGGCAAATAGCTTCTCTTACTGGCCGGGGCGCGGGGGGACCGAGCATCGGGCGTCATA
GGTCAAGGGTTTTCGTCATCCTGGTCGAGCGGCCCTGCTCGTACGAGCTCATTGGCATTATCACATAATG
AATTATACATTATATAAAGTAATGTGATTTCTTCGAAGAATATACTAAAAAATGAGCAGGCAAGATAAAC
GAAGGCAAAGATGGTGAGCAAGGGCGAGGAGCTGTTCACCGG

Mapped library reference sequence
ccgctacagggcgcgtcgcgccattcgccattcaggctgcgcaactgttgggaagggcgatcggtgcgggcctcttcgctattacgccag
ctggcgaaagggggatgtgctgcaaggcgattaagttgggtaacgccagggttttcccagtcacgacgttgtaaaacgacggccagtga
gcgcgcgtaatacgactcaCTATAGGGCGAATTGGGTACTGGACTGGATTGAAACGGGAACGAACGAGCCAC
GTGACGGGCTTCCTCTTAGTACGATACTTACGTTACTATTCAGTGATAACAATCACACTGTACGGCTGAT
CAGAGGAAGCTCGGTGGCAAATAGCTTCCTCTTACTGGCCGGGGCGCGGGGGGACCGAGCATCGGGC
GTCATAGGTCAAGGGTTTTCGTCATCCTGGTCGAGCGGCCNNNNTCGTACgagctcATTGGCATTATCACA
TAATGAATTATACATTATATAAAGTAATGTGATTTCTTCGAAGAATATACTAAAAAATGAGCAGGCAAGA
TAAACGAAGGCAAAGATGGTGAGCAAGGGCGAGGAGCTGTTCACCGGGGTGGTGCCCATCCTGGTCG
AGCTGGACGGCGACGTAAACGGCCACAAGTTCAGCGTGTCCGGCGAGGGCGAGGGCGATGCCACCTA
CGGCAAGCT

3) Ty5 Transposon inserted downstream of barcode and UMI in forward direction

3

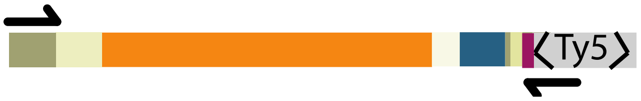

R1

CTATAGGGCGAATTGGGTACTGGACTGGATTTTTTCATTTTTTTTTTTTTCTGTAACCCGTACATGCCCTAG
AAAGTACGATACTTACGTTACTATTCAGTGATAGTAGGTGTCTGGGTGAACAGATCAAACCTGGCATCC
ACTAAATTTTCATTTTTTTTTTTTTATGCCGCTGGCATCCAGAAAGGCATCGTTTTTTTTTCTTTAAGGTGGG
CTGATCCTGGTCGAGCGGCCCTCATCGTACGGGCTCAT

R2

TTTTTTTTCTGTAACCCGTTCATGCCCTTGAAAGTTCGATTCTTACGTTACTATTCAGTGATAGTAGGTGTT
TGGGTTAACAGATCAAACCTGGCATCCACTAAATTTTCATTTTTTTTTTTTTATGCCGCTGGCATCCAGAAA
GGCATCGTTTTTTTTTCTTTAAGGTGGGCTGATCCTGGTCGAGCGGCCCTCATCGTACGAGCTCATTGGC
ATTTGTTGAATGTGATAACCCAAAAGCATGATATGGG

Ty5 inserted in forward direction downstream of barcode and UMI

Mapped library reference sequence

ccgctacagggcgcgtcgcgccattcgccattcaggctgcgcaactgttgggaagggcgatcggtgcgggcctcttcgctattacgccag
ctggcgaaagggggatgtgctgcaaggcgattaagttgggtaacgccagggttttcccagtcacgacgttgtaaaacgacggccagtga
gcgcgcgtaatacgactcaCTATAGGGCGAATTGGGTACTGGACTGGATTTTTTCATTTTTTTTTTTTTCTGTAACC
CGTACATGCCCTAGAAAGTACGATACTTACGTTACTATTCAGTGATAGTAGGTGTCTGGGTGAACAGATC
AAACCTGGCATCCACTAAATTTTCATTTTTTTTTTTTTATGCCGCTGGCATCCAGAAAAGCATCGTTTTTTTTT
CTTTAAGGTGGGCTGATCCTGGTCGAGCGGCCNNNNTCGTACgagctcATTGGCATTATCACATAATGAA
TTATACATTATATAAAGTAATGTGATTTCTTCGAAGAATATACTAAAAAATGAGCAGGCAAGATAAACGA
AGGCAAAGATGGTGAGCAAGGGCGAGGAGCTGTTCACCGGGGTGGTGCCCATCCTGGTCGAGCTGGA
CGGCGACGTAAACGGCCACAAGTTCAGCGTGTCCGGCGAGGGCGAGGGCGATGCCACCTACGGCAAG
CT

4) Ty5 Transposon inserted downstream of barcode and UMI in reverse direction

4

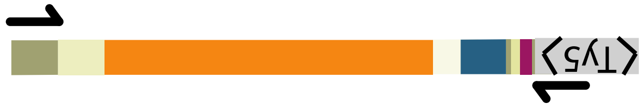

R1
CTATAGGGCGAATTGGGTACTGGACTGGATTTTTTCATTTTTTTTTTTCTGTAACCCGTACATGCCCTAGA
AAGTACGATACTTACGTTACTATTCAGTGATAGTAGGTGTCTGGGTGAACAGATCAAACCTGGCATCCAC
TAAATTTTCATTTTTTTTTTTTATGCCGCTGGCATCCAGAAAAGCATCGTTTTTTTTTCTTTAAGGTGGGCTG
ATCCTGGTCGAGCGGCCAAGTTCGTACGAGCTCATTG

R2
ATCAAACCTGGCATCCACTAAATTTTCATTTTTTTTTTTTATGCCGCTGGCATCCAGAAAAGCATCGTTTTTT
TTTCTTTAAGGTGGGCTGATCCTGGTCGAGCGGCCAAGTTCGTACGAGCTCATTGGCATTATCACATAAT
GAATTATACATTATATAAAGTAATGTGATTTCTTCGAAGAATATACTAAAAAATGAGCAGGCAAGATAAA
CGTGTTGACGTAGTGAATTACGATCTAGCTTATATGTC

Ty5 inserted in reverse direction downstream of barcode and UMI

Mapped library reference sequence
ccgctacagggcgcgtcgcgccattcgccattcaggctgcgcaactgttgggaagggcgatcggtgcgggcctcttcgctattacgccag
ctggcgaaagggggatgtgctgcaaggcgattaagttgggtaacgccagggttttcccagtcacgacgttgtaaaacgacggccagtga
gcgcgcgtaatacgactcaCTATAGGGCGAATTGGGTACTGGACTGGATTTTTTCATTTTTTTTTTTCTGTAACC
CGTACATGCCCTAGAAAGTACGATACTTACGTTACTATTCAGTGATAGTAGGTGTCTGGGTGAACAGATC
AAACCTGGCATCCACTAAATTTTCATTTTTTTTTTTTATGCCGCTGGCATCCAGAAAAGCATCGTTTTTTTTT
CTTTAAGGTGGGCTGATCCTGGTCGAGCGGCCNNNNTCGTACgagctcATTGGCATTATCACATAATGAA
TTATACATTATATAAAGTAATGTGATTTCTTCGAAGAATATACTAAAAAATGAGCAGGCAAGATAAACGA
AGGCAAAGATGGTGAGCAAGGGCGAGGAGCTGTTCACCGGGGTGGTGCCCATCCTGGTCGAGCTGGA
CGGCGACGTAAACGGCCACAAGTTCAGCGTGTCCGGCGAGGGCGAGGGCGATGCCACCTACGGCAAG
CT

5) Obtain normalization factor

To account for the variation of sequence representation in an experiment, we normalize the raw number of insertions to the fraction of each element in a library. Thus, we also performed a PCR that amplifies the inserted library without Ty5 transpositions.

R1

CTATAGGGCGAATTGGGTACTGGACTGGATTAGGACATTTTCATTTTTTTTTTTCTGTAACCCGTACATGC
CCTCAGTGTGGTTTTCATTTTTTTTTTTTTGGCGATCGAAGTAGGTGTCTGGGTGAACACATCCGTCCTGGC
ATCCACTAAACCATGTGAGCGTGCGGGAAAAGCTGGCATCCAGAAATTTTTTTTTCTTACTATCGGAGTG
GATCCTGGTCGAGCGGCCCTCCTCGTACGAGCTCATTG

R2

ACATCCGTCCTGGCATCCACTAAACCATGTGAGCGTGCGGGAAAAGCTGGCATCCAGAAATTTTTTTTTC
TTACTATCGGAGTGGATCCTGGTCGAGCGGCCCTCATCGTACGAGCTCATTGGCATTATCACATAATGAA
TTATACATTATATAAAGTAATGTGATTTCTTCGAAGAATATACTAAAAAATGAGCAGGCAAGATAAACGA
AGGCAAAGATGGTGAGCAAGGGCGAGGAGCTGTTCACCGG

ccgctacagggcgcgtcgcgccattcgccattcaggctgcgcaactgttgggaagggcgatcggtgcgggcctcttcgctattacgccag
ctggcgaaagggggatgtgctgcaaggcgattaagttgggtaacgccagggttttcccagtcacgacgttgtaaaacgacggccagtga
gcgcgcgtaatacgactcaCTATAGGGCGAATTGGGTACTGGACTGGATTAGGACATTTTCATTTTTTTTTTTCT
GTAACCCGTACATGCCCTCAGTGTGGGACGACGCTCAGTAGTTGGCGATCGAAGTAGGTGTCTGGGTG
AACACATCCGTCCTGGCATCCACTAAACCATGTGAGCGTGCGGGAATAAGCTGGCATCCAGAAATTTTTT
TTTCTTACTATCGGAGTGGATCCTGGTCGAGCGGCCNNNNTCGTACgagctcATTGGCATTATCACATAAT
GAATTATACATTATATAAAGTAATGTGATTTCTTCGAAGAATATACTAAAAAATGAGCAGGCAAGATAAA
CGAAGGCAAAGATGGTGAGCAAGGGCGAGGAGCTGTTCACCGGGGTGGTGCCCATCCTGGTCGAGCT
GGACGGCGACGTAAACGGCCACAAGTTCAGCGTGTCCGGCGAGGGCGAGGGCGATGCCACCTACGGC
AAGCT
